# Contributions of key countries, enterprises, and refineries to greenhouse gas emissions in global oil refining, 2000–2021

**DOI:** 10.1016/j.xinn.2022.100361

**Published:** 2022-12-08

**Authors:** Shijun Ma, Tianyang Lei, Jing Meng, Xi Liang, Dabo Guan

**Affiliations:** 1 Department of Earth System Sciences, Tsinghua University, Beijing 100080, China; 2 The Barlett School of Sustainable Construction, University College London, London WC1E 6BT, UK

**Keywords:** greenhouse gas emissions, global oil refineries, enterprises

## Abstract

The refining industry is the third-largest source of global greenhouse gas (GHG) emissions from stationary sources, so it is at the forefront of the energy transition and net zero pathways. The dynamics of contributors in this sector such as crucial countries, leading enterprises, and key emission processes are vital to identifying key GHG emitters and supporting targeted emission reduction, yet they are still poorly understood. Here, we established a global sub-refinery GHG emission dataset in a long time series based on life cycle method. Globally, cumulative GHG emissions from refineries reached approximately 34.1 gigatons (Gt) in the period 2000–2021 with an average annual increasing rate of 0.7%, dominated by the United States, EU27&UK, and China. In 2021, the top 20 countries with the largest GHG emissions of oil refining accounted for 83.9% of global emissions from refineries, compared with 79.5% in 2000. Moreover, over the past two decades, 53.9–57.0% of total GHG emissions came from the top 20 oil refining enterprises with the largest GHG emissions in 12 of these 20 countries. Retiring or installing mitigation technologies in the top 20% of refineries with the largest GHG emissions and refineries with GHG emissions of more than 0.1 Gt will reduce the level of GHG emissions by 38.0%–100.0% in these enterprises. Specifically, low-carbon technologies installed on furnaces and boilers as well as steam methane reforming will enable substantial GHG mitigation of more than 54.0% at the refining unit level. Therefore, our results suggest that policies targeting a relatively small number of super-emission contributors could significantly reduce GHG emissions from global oil refining.

## Public summary

• A global sub-refinery GHG emission dataset was developed.
• Key contributors to GHG emissions in the global oil refining industry were illustrated.
• Key process units for GHG emissions from global refineries were identified.

## Graphical abstract

**Figure:**
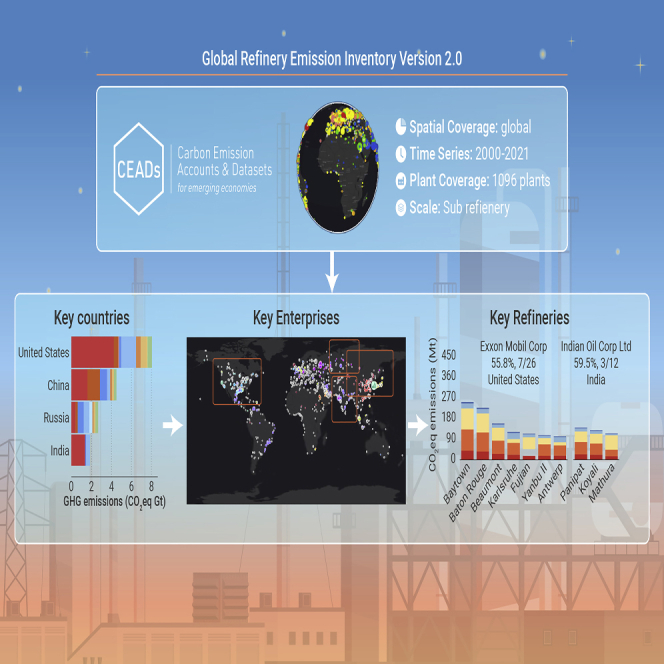

## Introduction

Global warming is one of the most critical environmental challenges humanity now faces.^1^ Many countries have set climate neutrality targets for limiting the global temperature increase to below 2°C, and even to avoid a 1.5°C increase, as required by the Paris Agreement,^2^^,^^3^^,^^4^ which stipulates net-zero CO_2_ emission in every sector by the second half of the 21st century.^3^^,^^5^ According to the Intergovernmental Panel on Climate Change (IPCC),^6^ from the beginning of 2020 the upper limit of CO_2_ absorbed in the atmosphere must be 1,170 gigatons (Gt) if the 2°C target is to be met, while the figure for the 1.5°C target is 400 Gt of CO_2_. Thus to mitigate global climate change, the energy transition urgently needs to accelerate the reduction of CO_2_ emissions in every sector.^7^

orldwide, the oil refining industry is the third-largest emitter of greenhouse gas (GHG) emissions from stationary sources, accounting for nearly 5% of global energy-sector GHG emissions in 2019.^8^^,^^9^ Moreover, from 2010 to 2018, GHG emissions in the oil refining sector surged by 24%.^10^ Although production in the global refining industry fell by 9% due to the COVID-19 pandemic, as population and GDP recover and continue to grow, emissions from this industry are sure to rebound and keep rising in the near future.^11^ Against the backdrop of growing pressure to reduce GHG emissions and the increasing demands of the oil refining industry, the identification of key contributors in the development and GHG emissions in that industry is urgently needed for targeted and adaptive carbon mitigation that will simultaneously meet aims for both carbon reduction and oil refined products.^12^

A publicly available, high-precision dataset with detailed and comprehensive information is key to providing targeted guidance for accurately estimating factory-level GHG emissions and formulating precise policies on emission reduction for the refining industry.^13^ Previous studies on decarbonizing the global oil refining industry have focused on GHG emissions at factory level^9^^,^^10^ and country level.^14^^,^^15^^,^^16^ However, these studies failed to factor in sub-refinery information, such as ownership structure and process units, which is the basis for identifying crucial countries, leading enterprises, and key emission processes with the largest GHG emissions, thus adopting policy-targeted reduction of the oil refining industry. These gaps in understanding of emissions from the oil refining industry will hinder the formulation of more accurate and effective decarbonization strategies. The systemized development of a high-quality refinery monitoring dataset is therefore important for pinning down information on, say, the state of operations and configuration types to help in addressing many of the analytic difficulties in understanding this industry’s GHG emissions.

Here, our study first integrated multiple data sources related to global oil refineries (including GlobalData, CEADs-GREIv1.0, S&P Capital IQ, and PRELIM; see details in Table S1) to establish a global sub-refinery GHG emission dataset in a long time series based on life cycle method, the Carbon Emission Accounts and Datasets-Global Refinery Emission Inventory Version 2.0 (CEADs-GREIv2.0) :https://www.ceads.net.cn/. GHG emission accounting of oil refining enterprises can provide an important reference for their emission reduction decisions, while the deployment of mitigation technologies based on process units needs the support of sub-refinery GHG emission data.^17^^,^^18^^,^^19^ Compared with the CEADs-GREIv1.0, additions to CEADs-GREIv2.0 include information on ownership structure, refining process, and type of refined crude oil, while GHG emissions are updated to 2021. We then used the CEADs-GREIv2.0 to identify the dynamics of key contributors to GHG emissions in the global oil refining industry over the past two decades from the perspectives of countries, enterprises, and refineries. Our findings explore the process unit, enterprise, national, and global levels of GHG emissions from the oil refining industry based on a life cycle perspective, which will lay a solid foundation for research on future mitigation pathways for this sector, especially the unit-based deployment of abatement technologies and mitigation strategies at plant or enterprise level.

## Results

### Dynamics of the GHG emission patterns in the global oil refining industry

**Figure 1:**
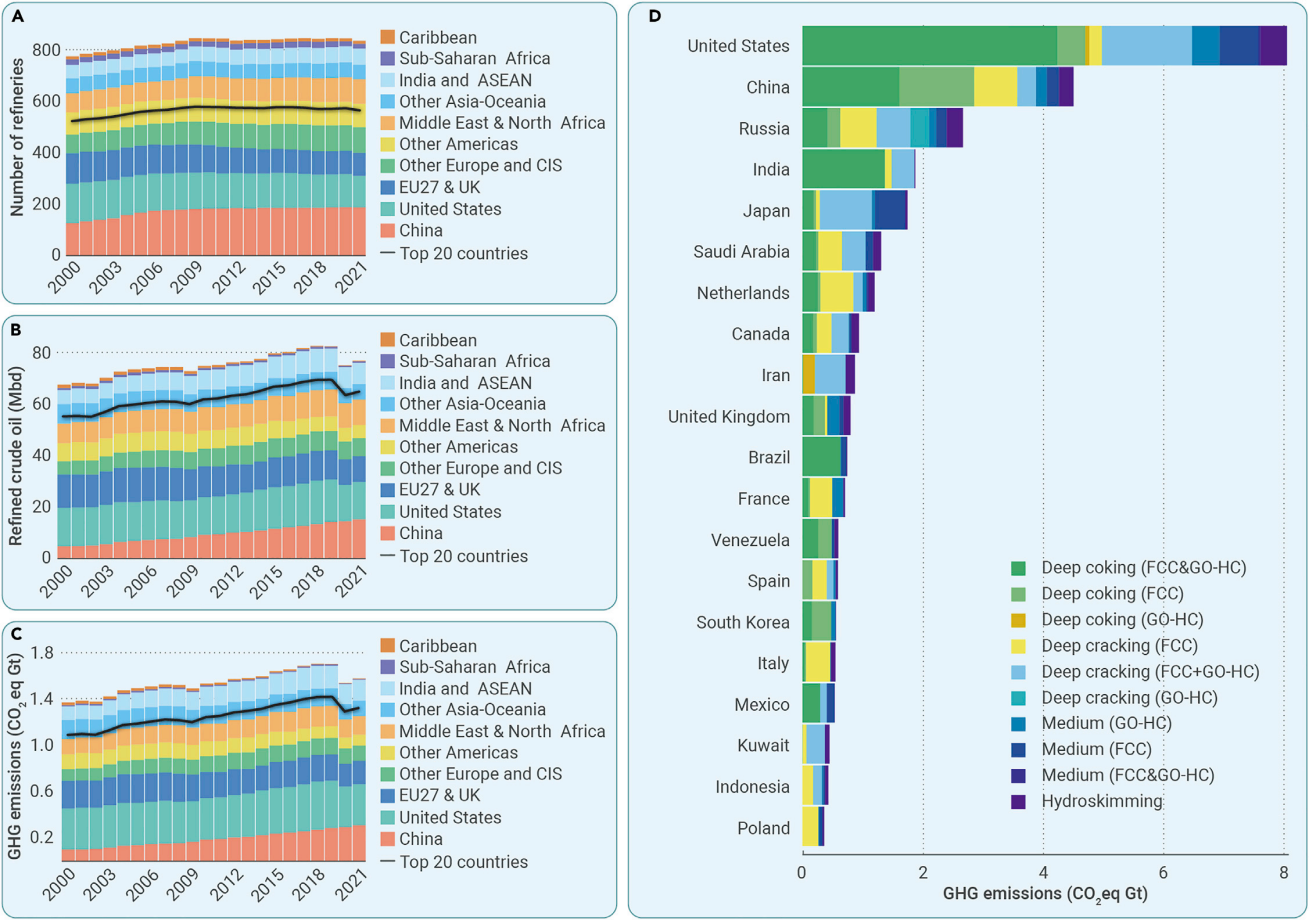
Trends of GHG emissions of global oil refineries from 2000 to 2021 by country, region, and refinery configuration type (A) Changes in number of refineries by regions. (B) Changes in the amount of crude oil refined in the oil refining industry by regions. (C) Trends of GHG emissions by regions. (D) GHG emissions in the top 20 countries with the highest GHG emissions from oil refining industry among all the countries worldwide, which will be named as the top 20 countries later. Note: 10 refinery technology types are included in this study, namely hydroskimming, medium conversion (FCC), medium conversion (GO-HC), medium conversion (FCC&GO-HC), deep coking (FCC), deep coking (GO-HC), deep coking (FCC&GO-HC), deep cracking (FCC), deep cracking (GO-HC), and deep cracking (FCC&GO-HC). Hydroskimming may contain basic process units such as desalter, atmospheric tower furnace, atmospheric tower, naphtha hydrotreater, isomerization unit, etc. In addition to the process units in the hydroskimming refinery, medium conversion (FCC), medium conversion (GO-HC), and medium conversion (FCC & GO-HC) also contains fluid catalytic cracking (FCC) and gas oil hydrocracker (GO-HC). Meanwhile, deep coking (FCC), deep coking (GO-HC), deep coking (FCC&GO-HC), deep cracking (FCC), deep cracking (GO-HC), and deep cracking (FCC&GO-HC) include not only terminal conversion units, but also deep conversion units such as coking or hydrocracking; see details in Table S2. The definition of the 10 regions in this study is shown in Figure S1.

From 2000 to 2021, the number of refineries globally rose from 739 to 839, with China, the United States, and the EU27&UK having the largest number, accounting for 47.9%–52.7% of the global total. Meanwhile, the amount of crude oil refined in the oil refining industry varied with the number of refineries in most regions (Figures 1A and 1B). The main driver of the surging demand for crude oil in the refining industry in China before 2007 was the increase in number of refineries (from 127 to 179), which shifted to a rise in average refinery capacity after 2007. In the United States, the number of refineries has declined from 155 to 123, but the amount of refined crude oil has remained roughly steady, fluctuating between 14.2 and 17.0 million barrels per day (Mbd). By contrast, volumes of crude oil refined in the other Americas, Sub-Saharan Africa, and the Caribbean have fallen significantly by 39.1% and 84.2%, respectively, because of decreasing average refinery capacity. Interestingly, the average refinery capacity of the top 20 countries is relatively high. In 2021, for example, these countries housed 67.1% of the world’s refineries, accounting for 80.7% of crude oil refined worldwide.

GHG emissions associated with global refineries are highly concentrated in specific regions (Figure 1C). Cumulative GHG emissions from refineries globally reached approximately 34.1 Gt between 2000 and 2021, with an average annual increasing rate of 0.7%. More than half of the total came from refineries in the United States, EU27&UK, and China, contributing 24.1%, 15.6%, and 12.6%, respectively. From 2000 to 2021, GHG emissions from Chinese refineries more than tripled, from 102.2 to 313.3 Mt, while the country’s share of GHG emissions from global oil refining industry has more than doubled from 7.4% to 19.7%. Similar rapid growth was also seen in GHG emissions from India and ASEAN (Association of Southeast Asian Nations) refineries. Meanwhile, the share of EU27&UK refineries' CO_2_eq emissions declined from 17.4% to 12.7%, and that of the United States gradually decreased from 25.9% in 2000 to 22.5% in 2021.

Specifically, countries with high GHG emissions usually also show high carbon intensity, which is high GHG emissions from refining a barrel of crude oil. (Figures 1D and S2). GHG emissions from the top 20 countries have become a growing proportion of the total such emissions from the global oil refining sector, accounting for 83.9% of the total in 2021, compared with 79.5% in 2000. The carbon intensity of the top 20 countries is 59.0 kg/bbl, much higher than the world average of 56.2 kg/bbl. Among these countries, the United States, India, Japan, South Korea, Italy, and Spain have relatively high carbon intensities, exceeding 60 kgCO_2_eq/bbl. This is because more than 80% of GHG emissions from the oil refining industry of these countries, barring Japan’s, came from deep conversion refineries. Countries at the lower end of the top 20 have comparatively low carbon intensity: the United Kingdom, for instance, ranks 15th among these nations and has a carbon emission intensity of just 42.9 kg/bbl.

### Trends in the GHG emissions of oil refining enterprises

**Figure 2:**
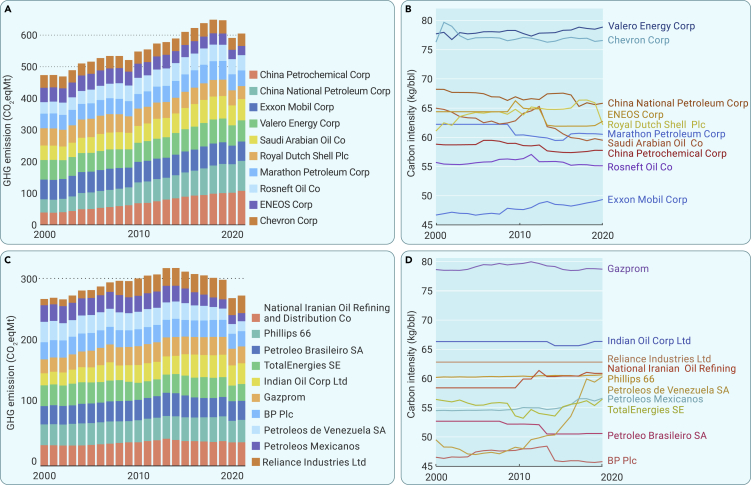
Dynamics in GHG emissions and the carbon intensity of global oil refining enterprises, 2000 to 2021 (A) Trends of GHG emissions in the top 10 enterprises with the highest GHG emissions from the oil refining industry among all the enterprises worldwide, which will be named as the top 10 enterprises later. (B) Trends of carbon intensity in the top 10 oil refining enterprises. (C) Trends of GHG emissions in the top 11–20 enterprises with the highest GHG emissions from oil refining industry among all the enterprises worldwide, which will be named as the top 11–20 enterprises later. (D) Trends of carbon intensity in the top 11–20 oil refining enterprises.

The 498 enterprises that make up the oil refining industry control 1,095 refineries globally. During 2000–2021, the top 20 enterprises—which comprise just 4% of the 498—dominated global oil refining production and GHG emissions with relatively high carbon intensity (Table S3). Among these top 20 enterprises, five belong to the United States, three to Russia, two to China, and two to India. In addition, Saudi Arabia, Iran, Brazil, the Netherlands, Venezuela, France, the United Kingdom, and Japan each have one enterprise in the top 20. Over the 2000–2021 period, GHG emissions from the top 10 of the 20 and from the remaining enterprises accounted for 33.8%–38.1% and 17.7%–20.5% of the global annual GHG emissions, with their average carbon emission intensity being 61.4–62.4 and 56.3–59.1 kgCO_2_eq/bbl, respectively (Figure 2).

Most of the top 20 enterprises came from a few developed countries, or countries with large refining capacities controlled by a local oil refining industry. GHG emissions from oil refining in developing countries are dominated by government-owned enterprises (Table S4). For example, up to 67.0% of GHG emissions in Chinese refineries came from China National Petroleum Corporation and China Petrochemical Corporation. In Iran, the National Iranian Oil Refining and Distribution Company, a government-owned enterprise that essentially controls the country’s entire refining industry, is responsible for up to 98.4% of that country’s related GHG emissions. BP plc, by contrast, is a special case: an entirely foreign investment enterprise, with all its GHG emissions occurring overseas (Figure S3).

There are three GHG emission patterns for the top 20 enterprises in the last two decades, including stable emissions, rapidly growing emissions, and recessionary emissions. GHG emissions from the oil refining industry in some developed countries show a high and steady trend; cases in point include Valero Energy Corp, Marathon Petroleum Corporation, and Phillips 66 (Figures 2A and 2C), indicating a stable, heavy demand for petroleum products in the United States. Meanwhile, GHG emissions from government-owned oil refining enterprises in emerging countries grew significantly. For example, GHG emissions from the China National Petroleum Corporation and China Petrochemical Corporation had both more than doubled from 2000 to 2019, soaring from 39.2 and 41.7 Mt in 2000 to 100.4 and 92.1 Mt in 2019, respectively. Such an apparent increase also showed in GHG emissions from the Saudi Arabian Oil Company. What drove the boom in GHG emissions from the oil refining industry in these emerging countries was the construction of modern major refineries and the growing domestic demand for refined petroleum products. Conversely, the ENEOS Corporation in Japan declined significantly: its GHG emissions in 2019 were only 78.1% of those in 2000.^20^ The carbon intensity of ENEOS Corp also fell by 4% (Figure 2B), indicating shrinkage in the corporation’s oil refining capacity, which was caused in turn by a decline in the countries' petroleum demand^21^ and by the adjustment of energy structure and energy-saving plans in Japan. A similar sharp decline in GHG emissions could be found in Petroleos de Venezuela SA and Petroleos Mexican OS, from 33.6 to 27.4 Mt in 2000 to 18.6 and 15.2 Mt in 2019, respectively. However, during the same period the carbon intensity of these two enterprises rose by 21.0% and 4.7%, respectively (Figure 2D), due to production cuts and decommissioning of refineries caused by the economic downturn and political turmoil.^22^

Compared with ENEOS Corp, Petroleos de Venezuela SA, and Petroleos Mexican OS, the carbon intensity in most of the top 20 enterprises has remained relatively constant over the past two decades, such as China National Petroleum Corporation and China Petrochemical Corporation. In addition, the top 10 enterprises have higher carbon intensity than the top 11–20 ones: except for Exxon Mobil Corporation, the average carbon intensity of those top 10 is higher than 55 kg CO_2_eq/bbl, while only half of the top 11–20 enterprises have an average carbon intensity higher than 55 kg CO_2_eq/bbl (Figures 2B and 2D). This could mean that enterprises with high GHG emissions also have high carbon intensity.

### GHG emissions in the oil refining processes

**Figure 3:**
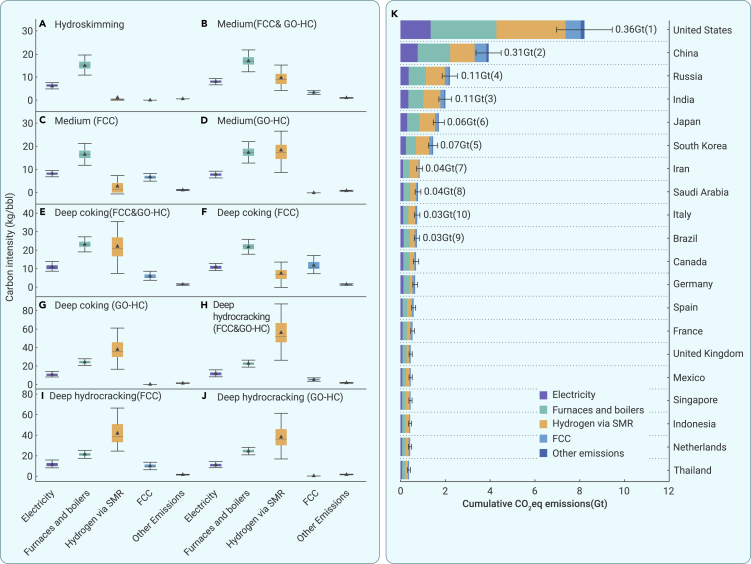
GHG emissions based on process units GHG emissions based on process units in (A) hydroskimming refineries, (B) medium conversion refineries (FCC), (C) medium conversion refineries (GO-HC), (D) medium conversion refineries (FCC&GO-HC), (E) Deep coking refineries (FCC), (F) deep coking refineries (GO-HC), (G) deep coking refineries (FCC&GO-HC), (H) deep hydrocracking refineries (FCC), (I) deep hydrocracking refineries (GO-HC), and (J) deep hydrocracking refineries (FCC&GO-HC). (K) Cumulative GHG emissions based on process units in the top 20 countries. The number in Figure 2K represents GHG emissions from the refining industry in the top 20 countries in 2021.

Currently, the installation of abatement facilities is focused on four main processes in refining: electricity, furnaces and boilers, fluid catalytic cracking (FCC), and steam methane reforming (SMR).^23^ We integrated the remaining process emissions such as support services emissions and releasing from managed wastes into other emissions. Therefore, based on the life cycle method and the existing crude oil sample database, we estimated and summarized the GHG emissions of five process units in 10 refinery configurations, as shown in Figure 3A. GHG emissions from furnaces and boilers and electricity in hydroskimming refineries account for 66.2% and 26.5%, respectively, of total GHG emissions. However, in medium conversion refineries, FCC or gas oil hydrocracking (GO-HC) contributes a considerable amount of GHG emissions, so these have emerged as the second-largest GHG emission process in refineries (Figures 3B–3D). Compared with hydroskimming refineries, the proportion of GHG emissions generated by furnaces and boilers is relatively small, accounting for 40.3%, 32.5%, and 36.3% in deep coking refineries (FCC), deep coking refineries (GO-HC), and deep coking refineries (FCC&GO-HC), respectively (Figures 3E–3G). Hydrogen via SMR is the largest GHG emitter in deep hydrocracking refineries, accounting for 48.1%, 66.4%, and 58.0% in deep hydrocracking refineries (FCC), deep hydrocracking refineries (GO-HC), and deep hydrocracking refineries (FCC&GO-HC), respectively (Figures 3H–3J).

In the top 20 countries, GHGs from furnaces and boilers accounted for 30.1%–43.5% of total cumulative refinery GHG emissions from 2000 to 2021 (Figure 3K), while this proportion increased to 47.0%–67.2% in the 20 countries with the lowest emissions from the refining sector (Figure S4). This is mainly due to the relatively high proportion of deep conversion refineries that have relatively low GHG emissions from furnaces and boilers in the top 20 countries. For example, deep conversion refineries in the United States accounted for 81.1% of total refinery production in 2021, resulting in 35.8% of GHG emissions from heating processes, while there was all simple hydroskimming refineries in Laos leading to 65% of GHG emissions from heating processes (Figure S5). Hydrogen via SMR was also a crucial GHG emission processes in oil refineries in top 20 countries, accounting for 17.7%–49.2% (Figure 3). This is because medium and heavy conversion refineries require large amounts of hydrogen to convert heavy oil into light oil. Moreover, electricity was the third-largest source of GHG emissions from refineries, contributing 13.1%–20.7% of the total emissions in the top 20 countries.

**Figure 4:**
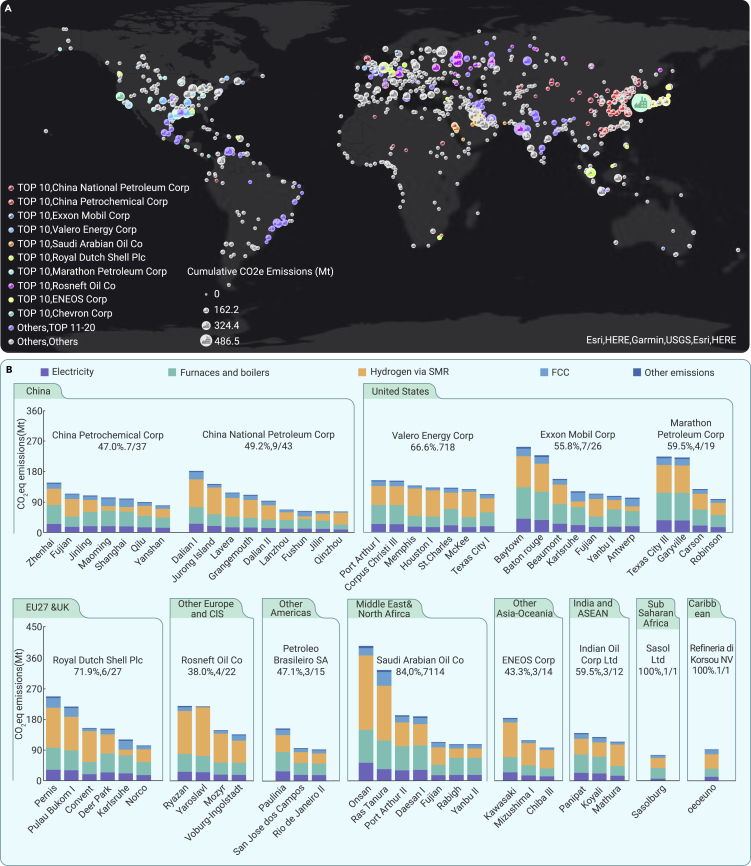
Cumulative process GHG emissions of crucial enterprises from 2000 to 2021 (A) Location, enterprises, and cumulative GHG emissions of 1,405 oil refineries worldwide. Color of the point shows the refining enterprises to which the refinery belongs. The size of points indicates the cumulative GHG emissions size (≤162.2 Mt, ≤324.4 Mt, ≤486.5 Mt). (B) The process GHG emissions of top refineries in the enterprise with the largest GHG emissions in each region. The notes in Figure 4B respectively show the proportion of GHG emissions of important refineries in the enterprise and the proportion of key refineries in the total refineries in the enterprise. The top 20% refineries with the largest GHG emissions in an enterprise or refineries with GHG emissions more than 0.1 Gt controlled by an enterprise were named as top refineries. The process GHG emissions of top refineries in the other top 20 enterprises are shown in Figure S6.

The process emissions in the top refineries controlled by the enterprise with the largest GHG emissions in each region were analyzed (Figure 4). Notably, there are more than 20% of their refineries with GHG emissions exceeding 0.1 Gt in five enterprises including Valero Energy Corporation, Exxon Mobil Corporation, Royal Dutch Shell Plc, Saudi Arabian Oil Company, and Indian Oil Corporation Ltd. The GHG emissions of these refineries accounted for 55.8%–84.0% of total such emissions in these five enterprises, respectively. The top 20% refineries with the largest GHG emissions accounted for 38.0%–59.5% of total GHG emissions in another six enterprises including China Petrochemical Corporation, China National Petroleum Corporation, Marathon Petroleum Corporation, Resneft Company, Petroleum Brasilero SA, and ENEOS Corporation. We further analyzed the GHG emissions of refining processing units within the top refineries controlled by these enterprises, within the context of reducing emissions (Figure 4B). The four main emissions sources associated with processing—electricity, furnaces and boilers, hydrogen via SMR and FCC—accounted for 96.9%–98.8% of total GHG emissions. However, due to the difference in configuration types of refineries, low-carbon deployment pathways for each refinery are not straightforward. For example, hydrogen via SMR is the largest GHG emission source in the Fujian refinery, while heat and steam constitute the largest in the Maoming refinery. Plants will inevitably need to factor the type of source into their mitigation strategies, for instance in prioritizing which refining units must install GHG mitigation technologies. A refinery GHG emission inventory based on the life cycle method can thus provide basic data support for the deployment of each refinery emission reduction technology.

## Discussion

This study built CEADs-GREI v2.0 as a global sub-refinery GHG emission database based on the life cycle method. This expands on an earlier version, CEADs-GREI,^10^ by adding and exploring GHG emissions from the sub-refinery level. Moreover, it identifies three kinds of major contributors to refinery GHG emissions: highest-emitting countries to reduce GHG emissions, enterprises with crucial responsibilities, and key refinery processes, thus providing more detailed basic data support for targeted GHG emissions reduction in the oil refining industry. We estimated the current global GHG emissions from refineries in successive years, which will depend on the type and output of refineries, and we did not consider the implementation of mitigation strategies in the oil refining sector. Previous studies on GHG emissions from global oil refineries based on point sources focused on the comparison of carbon intensity and GHG emissions among countries.^9^^,^^10^ This study is the first to examine national process unit-based GHG emissions of the oil refining industry from a sub-refinery scale, highlighting the important contribution of key process units in a few countries to GHG emissions from global oil refineries. Moreover, we also highlighted the importance of a small number of major enterprises and oil refineries in global GHG emissions from the oil refining industry.

### Key mitigation strategies for countries and enterprises

The top 20 countries with the highest GHG emissions from the global oil refining industry are at the core of GHG emission reduction in each region (Table S5), while the 20 enterprises with the highest GHG emissions are located in these 20 countries, which play an important role in the GHG emissions of the refining industry in these countries (Figure 2 and Table S3). GHG emissions from refineries in energy-leading developed countries like the United States have been both huge and stable. These countries should take proactive measures to reduce GHG emissions in the oil refining sector, such as energy transformation to achieve refinery production reduction or decommissioning or the deployment of carbon capture, utilization, and storage (CCUS).^8^^,^^11^ Another key point is that the largest enterprises with the largest GHG emissions play a significant role in GHG emissions from refineries in such countries and are privately owned. For example, five of the top 20 enterprises in the United States, Valero Energy, Phillips 66, Marathon Petroleum, ExxonMobil, and Chevron, are all non-government-owned (Table S4), yet they accounted for 53.6% of GHG emissions from the US oil refining industry as a whole. Elements of the free market economic system make it difficult for the government to control these enterprises strictly.^18^ Thus effective emission reductions in this sector in such countries depends on the independent actions of the companies themselves.

In some developed countries, including the United Kingdom and Japan, the demand for petroleum products is declining even as an energy transformation is being actively promoted. These nations are likely to continue reducing GHG emissions from the oil refining industry in the future.^10^ Privately owned oil refining companies also dominated GHG emissions in these countries. Given the industry’s downturn in them, there may be a greater need for state financial subsidies to encourage companies to install mitigation facilities for their refineries in these countries. By contrast, emerging countries like China and India need an increasing volume of fossil fuels such as oil products to support their economic growth, thus increasing the number of refineries and expanding their refinery capacity to meet demand (Figures 1A and 1B). As a result, these countries become the main driver of increased GHG emissions from the global refining industry.^24^^,^^25^ These countries should install emission-reduction facilities in their refineries and take specific low-carbon actions such as embarking on an energy transformation to control demand for refinery products while also meeting the climate target. Government-owned enterprises, including the China National Petroleum Corporation, China Petrochemical Corporation, and Indian Oil Corporation Ltd, dominated national GHG emissions from refineries in these nations.^26^ Government policy intervention may thus push them to achieve effective mitigation measures. GHG emissions from the refining industry in some developing countries such as Venezuela, however, have been declining due to underinvestment caused by the financial crisis and political and domestic turmoil.^22^ It is thus difficult to demand that they reduce GHG emissions in the near term.

National and corporate GHG emission reduction will eventually be implemented through the installation of mitigation facilities in crucial refineries. The carbon intensity of most of the top 20 enterprises remained stable or varied slightly over time, mainly because the new construction and decommissioning of a few refineries did not lead to drastic changes in the refinery configuration structure of these enterprises. Thus, refineries with rates of GHG emissions in the top 20% and those with GHG emissions greater than 0.1 Gt have consistently dominated the change in carbon intensity of these enterprises and have accounted for 54.0% of GHG emissions of the top 20 enterprises over the past two decades. The deployment of abatement facilities in these refineries will significantly reduce GHG emissions from the refining industry in the top 20 companies. For example, achieving net-zero carbon emissions from seven refineries with largest carbon emissions in China Petrochemical Corporation would reduce its GHG emissions by 47% (Figure 4). Moreover, our results show that furnaces and boilers as well as hydrogen via SMR are process units with the highest GHG emissions at the refinery, accounting for at least 54.0% of total refinery emissions, similar to findings in previous studies.^27^^,^^28^ Prioritizing the installation of carbon mitigation technologies according to the configuration of the refinery’s processing units is essential if each refinery is to achieve GHG reductions. Furthermore, the significant disparity in the CO_2_ content of exhaust gas and the aggregation of the same process unit in refineries will further affect the difficulty and cost of implementing CO_2_ capture and storage (CCS) in these processes.^29^ For example, due to the ubiquity of furnaces and boilers in refineries causing the difficulty of collecting GHG in these units, post-combustion CCS at these unit will be limited; exhaust gas generated by hydrogen production, however, has high CO_2_ purity (20%–99%), which could be easier to capture at a lower cost.^23^ Therefore, compared with the top 11–20 countries, it is easier for the top 10 countries to reduce GHG emissions at a lower economic cost early on because of the small proportion of GHG emissions from furnaces and boilers. Analyzing GHG emissions during the refining process in key refineries will support the targeted implementation of emission reduction technologies, and thus drive countries and enterprises to take action on mitigation.^9^^,^^30^

### The uncertainty of estimating GHG emissions from global refineries

The uncertainty associated with estimating GHG emissions from refineries is mainly caused by the type of refinery configuration, the type of crude oil, and so on.^31^ We assigned 148 crude oil samples to three types of refineries based on the rules laid out in Table S6. However, this rough rule brought great uncertainty to the estimation of GHG emissions from refineries with different configurations in our study. The uncertainty of estimating GHG emissions from the global oil refining industry is shown in Figure S7. The GHG emissions from the global refining industry estimated in this study increased from 1.38 ± 0.21 Gt in 2000 to 1.59 ± 0.24 Gt in 2021. To test the reliability of the data, we also compared our results with previous studies. Jing et al.^9^ and Lei et al.^10^ reported global refining sector GHG emissions of 1.2 and 1.3 Gt in 2015 and 2018, respectively, while the GHG emissions calculated in this study in the same years were 38% and 32% higher than those in previous studies (Table S7). This may be because there is a wider range of data, covering 1,195 refineries in operation in 121 countries in the CEADS-GREI v2.0 database.

### The demand for a near-real-time refinery GHG emission database

In the future, we hope to be able to monitor the GHG emission reduction of global refineries in near real time in CEADS-GREI v3.0, with more refined timescale GHG emissions at the month level and smaller time lag of GHG emission report from oil refineries worldwide. A near-real-time refinery GHG emission database could also supply detailed, accurate data support for the oil refining industry to achieve energy transition and GHG emission reduction targets, especially in the wake of major natural and social disasters, such as COVID-19, the 2008 economic meltdown, and so on.^32^ Meanwhile, we plan to collect information on mitigation technologies that have been installed or have been planned to be installed in global refineries before 2060 to monitor and supervise the realization of GHG emission reduction targets worldwide.

## Materials and methods

### New CEADs-GREI

On the basis of the original database, we integrated multiple datasets and established the CEADs-GREI v2.0, whose data accuracy reached the sub-refinery level for the first time.^10^ (See Table S1 for data sources and basic database information.) Compared with the original database, the new version 2.0 mainly refined three parameters: (1) ownership structure of each refinery (including the top seven shareholders and their shares); (2) all industrial processes of the refinery (including process units, service life, and crude oil refining capacity of process units for successive years); and (3) three refinery configuration types, refined into 10 refinery configuration types. The crude oil refining amount (CORA) of consecutive years and months is obtained through standardization:

We estimated the CORA of global refineries from January 2000 to June 2021 using the following equation.

(Equation 1) $$P_{m,n,Y}={\text{CA}}_{m,n,Y}/{\text{CA}}_{n,Y}{*P}_{n,Y}*1000*365\text{,}$$

where P_m,n,Y_ represents the CORA of refinery *m* of country *n* in year *Y*, bbl; CA_m,n,Y_ represents daily crude oil refining capacity (CORC) of refinery *m* of country *n* in year *Y*, thousands of barrels per day (kbd); CA_n,Y_ represents daily CORC of country *n* in year *Y*, kbd; P_n,Y_ represents daily CORA of country *n* in year *Y*, kbd. CA_m,n,Y_ and CA_n,Y_ come from CEADs-GREI database: https://www.ceads.net.cn/. P_n,Y_ comes from the BP Statistical Review of World Energy 2021.^33^

### Construction of GHG emissions database based on refinery industrial process unit

This study used PRELIM version 1.5. PRELIM is the first open-source tool capable of estimating GHG emissions and energy use in different refinery types, which can be allocated to all process units by using the life cycle method. The system boundary of the PRELIM model is the boundary of the refinery itself, including all major process units as well as all energy and hydrogen supplied to the refinery.^9^ The functional unit in PRELIM is 1 bbl input crude oil. PRELIM can simulate 10 refinery configurations based on the complexity of refining and how they deal with heavy fractions of crude oil. The model details of this model could be found in Bergerson et al. (2020).^34^ Therefore, this study can break refinery configurations down into 10 types, compared with four types in the CEADs-GREI v1.0.^10^ Detailed characteristics of the ten configurations of refineries are shown in Table S2. Details of basic background parameters set of PRELIM model in this study can be found in Table S8. PRELIM version 1.5 contains detailed parameters of 148 crude oil samples available in various regions of the world.

Based on the database, we used the PRELIM model to calculate the life cycle GHG emissions of each oil sample under different refinery configurations, and we used the average to estimate the total GHG emissions of each refinery configuration and each industrial process unit, with standard deviation representing their uncertainty (Equations 2 and 3).^35^

(Equation 2) $${\text{Cmean}}_{b,c}=\sum_{a=1}^{n}C_{a,b,c}/n$$

(Equation 3) $${\text{Csd}}_{b,c}=\sqrt{\sum_{a=1}^{n}{\left(C_{a,b,c}-{\text{Cmean}}_{b,c}\right)}^{2}}\text{,}$$

where Cmean_b,c_ represents the average GHG emissions of 1 kg crude oil sample of the process *c* in the refinery of type *b*, kg/bbl; C_a,b,c_ represents GHG emissions of 1 kg crude oil sample *a* of the process *c* in the refinery of type *b*, kg/bbl. *b* Includes 10 refinery types; *c* contains five kinds of industrial process unit, including heaters and boilers, hydrogen production via SMR, utilities, FCC, and subprocesses. Csd_b,c_ represents the standard deviation of GHG emissions of 1 kg crude oil sample in process *c* in the refinery of type *b*, kg/bbl.

### GHG emissions accounting

This study analyzed sub-refinery GHG emissions from three aspects (industrial process, more refined timescale, and ownership structure). The total GHG emissions per unit of crude oil and per industrial process in different refinery configurations were obtained by using the PRELIM model. The CORA of a single refinery in consecutive years was obtained through standardization and dimension reduction. Therefore, we estimated annual GHG emissions and industrial process GHG emissions using Equation 4. The GHG emissions of holding companies were obtained by splitting and summarizing the GHG emissions of a single refinery in proportion. The ownership structure we used is the data of the enterprises in 2021.

(Equation 4) $${\text{CE}}_{m,n,Y}=\sum_{c=1}^{5}{\text{Cmean}}_{b,c}{*P}_{m,n,Y}\text{,}$$

where CE_m,n,Y_ represents refinery GHG emissions of refinery *m* of country *n* in year *Y*, kg, which is calculated at sub-refinery scale.

## Lead contact website

Jing Meng: https://www.ucl.ac.uk/bartlett/construction/people/dr-jing-meng.

Dabo Guan: http://faculty.dess.tsinghua.edu.cn/guandabo/zh_CN/index.htm.

## Supplemental information

Document S1. Figures S1–S7 and Tables S1–S8

Document S2. Article plus supplemental information
