# Supplementary material for: Contributions of key countries, enterprises, and refineries to greenhouse gas emissions in global oil refining, 2000–2021
Source: Innovation (Camb). 2022 Dec 8;4(1):100361. doi: 10.1016/j.xinn.2022.100361 (PMC9804246; doi:10.1016/j.xinn.2022.100361)
Supplement: Document S2. Article plus supplemental information [file mmc2.pdf]

REPORT

Contributions of key countries, enterprises, and refineries to greenhouse gas emissions in global oil refining, 2000–2021

Shijun Ma,[1,2,3] Tianyang Lei,[1,3] Jing Meng,[2,*] Xi Liang,[2] and Dabo Guan[1,2,*]

*Correspondence: jing.j.meng@ucl.ac.uk (J.M.); guandabo@tsinghua.edu.cn (D.G.)

Received: June 2, 2022; Accepted: December 5, 2022; Published Online: December 8, 2022; https://doi.org/10.1016/j.xinn.2022.100361

© 2022 The Authors. This is an open access article under the CC BY license (http://creativecommons.org/licenses/by/4.0/).

GRAPHICAL ABSTRACT

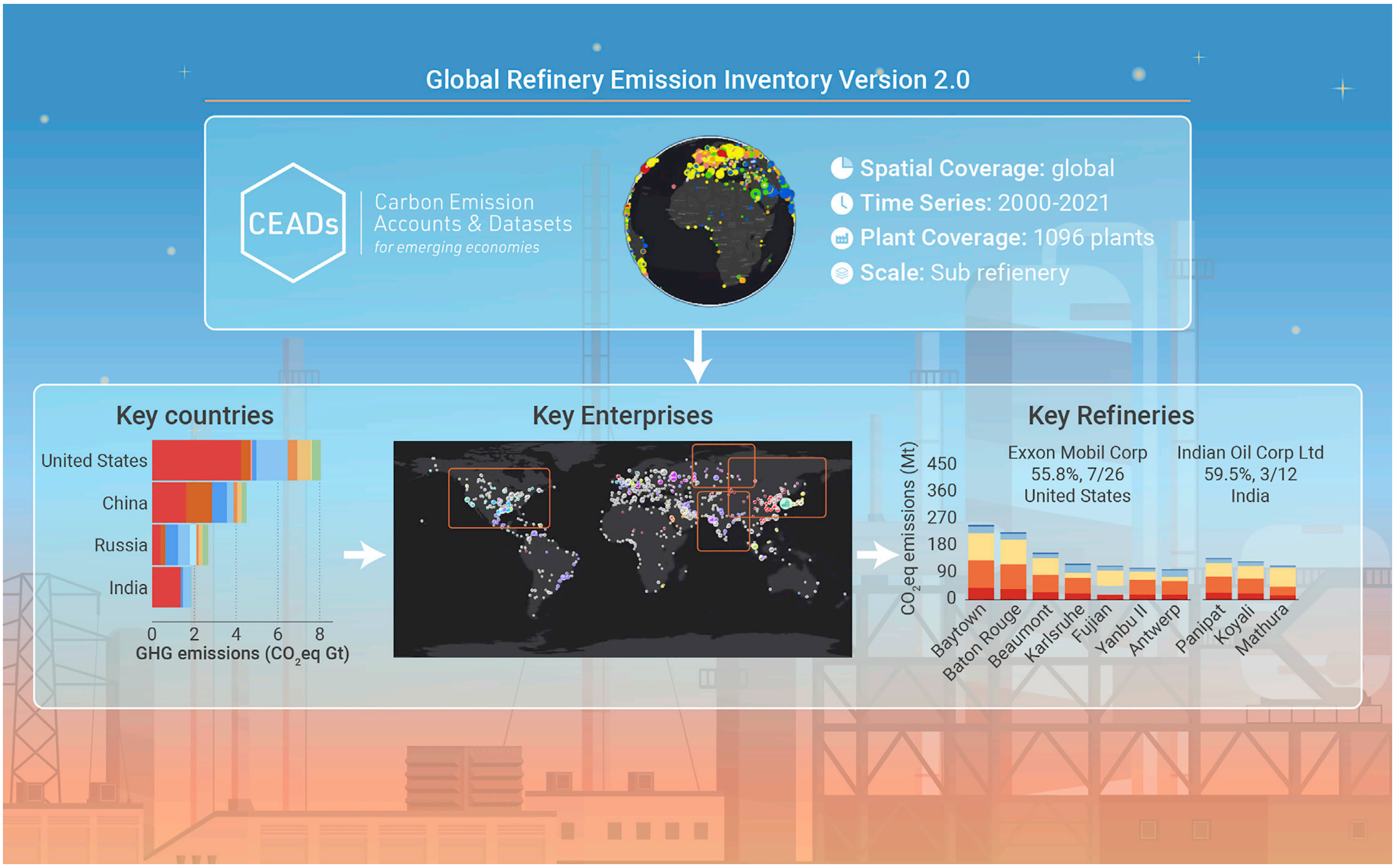

PUBLIC SUMMARY

- A global sub-refinery GHG emission dataset was developed.
- Key contributors to GHG emissions in the global oil refining industry were illustrated.
- Key process units for GHG emissions from global refineries were identified.

Contributions of key countries, enterprises, and refineries to greenhouse gas emissions in global oil refining, 2000–2021

Shijun Ma,[1,2,3] Tianyang Lei,[1,3] Jing Meng,[2,*] Xi Liang,[2] and Dabo Guan[1,2,*]
[1]Department of Earth System Sciences, Tsinghua University, Beijing 100080, China
[2]The Barlett School of Sustainable Construction, University College London, London WC1E 6BT, UK
[3]These authors contributed equally
*Correspondence: jing.j.meng@ucl.ac.uk (J.M.); guandabo@tsinghua.edu.cn (D.G.)
Received: June 2, 2022; Accepted: December 5, 2022; Published Online: December 8, 2022; https://doi.org/10.1016/j.xinn.2022.100361
© 2022 The Authors. This is an open access article under the CC BY license (http://creativecommons.org/licenses/by/4.0/).
Citation: Ma S., Lei T., Meng J., et al., (2023). Contributions of key countries, enterprises, and refineries to greenhouse gas emissions in global oil refining, 2000–2021. The Innovation **4(1)**, 100361.

The refining industry is the third-largest source of global greenhouse gas (GHG) emissions from stationary sources, so it is at the forefront of the energy transition and net zero pathways. The dynamics of contributors in this sector such as crucial countries, leading enterprises, and key emission processes are vital to identifying key GHG emitters and supporting targeted emission reduction, yet they are still poorly understood. Here, we established a global sub-refinery GHG emission dataset in a long time series based on life cycle method. Globally, cumulative GHG emissions from refineries reached approximately 34.1 gigatons (Gt) in the period 2000–2021 with an average annual increasing rate of 0.7%, dominated by the United States, EU27&UK, and China. In 2021, the top 20 countries with the largest GHG emissions of oil refining accounted for 83.9% of global emissions from refineries, compared with 79.5% in 2000. Moreover, over the past two decades, 53.9–57.0% of total GHG emissions came from the top 20 oil refining enterprises with the largest GHG emissions in 12 of these 20 countries. Retiring or installing mitigation technologies in the top 20% of refineries with the largest GHG emissions and refineries with GHG emissions of more than 0.1 Gt will reduce the level of GHG emissions by 38.0%–100.0% in these enterprises. Specifically, low-carbon technologies installed on furnaces and boilers as well as steam methane reforming will enable substantial GHG mitigation of more than 54.0% at the refining unit level. Therefore, our results suggest that policies targeting a relatively small number of super-emission contributors could significantly reduce GHG emissions from global oil refining.

INTRODUCTION

Global warming is one of the most critical environmental challenges humanity now faces.[1] Many countries have set climate neutrality targets for limiting the global temperature increase to below 2°C, and even to avoid a 1.5°C increase, as required by the Paris Agreement,[2–4] which stipulates net-zero CO_2 emission in every sector by the second half of the 21st century.[3,5] According to the Intergovernmental Panel on Climate Change (IPCC),[6] from the beginning of 2020 the upper limit of CO_2 absorbed in the atmosphere must be 1,170 gigatons (Gt) if the 2°C target is to be met, while the figure for the 1.5°C target is 400 Gt of CO_2. Thus to mitigate global climate change, the energy transition urgently needs to accelerate the reduction of CO_2 emissions in every sector.[7]

orldwide, the oil refining industry is the third-largest emitter of greenhouse gas (GHG) emissions from stationary sources, accounting for nearly 5% of global energy-sector GHG emissions in 2019.[8,9] Moreover, from 2010 to 2018, GHG emissions in the oil refining sector surged by 24%.[10] Although production in the global refining industry fell by 9% due to the COVID-19 pandemic, as population and GDP recover and continue to grow, emissions from this industry are sure to rebound and keep rising in the near future.[11] Against the backdrop of growing pressure to reduce GHG emissions and the increasing demands of the oil refining industry, the identification of key contributors in the development and GHG emissions in that industry is urgently needed for targeted and adaptive carbon mitigation that will simultaneously meet aims for both carbon reduction and oil refined products.[12]

A publicly available, high-precision dataset with detailed and comprehensive information is key to providing targeted guidance for accurately estimating factory-level GHG emissions and formulating precise policies on emission reduction for the refining industry.[13] Previous studies on decarbonizing the global oil refining industry have focused on GHG emissions at factory level[9,10] and country level.[14–16] However, these studies failed to factor in sub-refinery information, such as ownership structure and process units, which is the basis for identifying crucial countries, leading enterprises, and key emission processes with the largest GHG emissions, thus adopting policy-targeted reduction of the oil refining industry. These gaps in understanding of emissions from the oil refining industry will hinder the formulation of more accurate and effective decarbonization strategies. The systemized development of a high-quality refinery monitoring dataset is therefore important for pinning down information on, say, the state of operations and configuration types to help in addressing many of the analytic difficulties in understanding this industry's GHG emissions.

Here, our study first integrated multiple data sources related to global oil refineries (including GlobalData, CEADs-GREIv1.0, S&P Capital IQ, and PRELIM; see details in Table S1) to establish a global sub-refinery GHG emission dataset in a long time series based on life cycle method, the Carbon Emission Accounts and Datasets-Global Refinery Emission Inventory Version 2.0 (CEADs-GREIv2.0) :https://www.ceads.net.cn/. GHG emission accounting of oil refining enterprises can provide an important reference for their emission reduction decisions, while the deployment of mitigation technologies based on process units needs the support of sub-refinery GHG emission data.[17–19] Compared with the CEADs-GREIv1.0, additions to CEADs-GREIv2.0 include information on ownership structure, refining process, and type of refined crude oil, while GHG emissions are updated to 2021. We then used the CEADs-GREIv2.0 to identify the dynamics of key contributors to GHG emissions in the global oil refining industry over the past two decades from the perspectives of countries, enterprises, and refineries. Our findings explore the process unit, enterprise, national, and global levels of GHG emissions from the oil refining industry based on a life cycle perspective, which will lay a solid foundation for research on future mitigation pathways for this sector, especially the unit-based deployment of abatement technologies and mitigation strategies at plant or enterprise level.

RESULTS

Dynamics of the GHG emission patterns in the global oil refining industry

From 2000 to 2021, the number of refineries globally rose from 739 to 839, with China, the United States, and the EU27&UK having the largest number, accounting for 47.9%–52.7% of the global total. Meanwhile, the amount of crude oil refined in the oil refining industry varied with the number of refineries in most regions (Figures 1A and 1B). The main driver of the surging demand for crude oil in the refining industry in China before 2007 was the increase in number of refineries (from 127 to 179), which shifted to a rise in average refinery capacity after 2007. In the United States, the number of refineries has declined from 155 to 123, but the amount of refined crude oil has remained roughly steady, fluctuating between 14.2 and 17.0 million barrels per day (Mbd). By contrast, volumes of crude oil refined in the other Americas, Sub-Saharan Africa, and the Caribbean have fallen significantly by 39.1% and 84.2%, respectively, because of decreasing average refinery capacity. Interestingly, the average refinery capacity of the top 20 countries is relatively high. In 2021, for example, these countries housed 67.1% of the world's refineries, accounting for 80.7% of crude oil refined worldwide.

GHG emissions associated with global refineries are highly concentrated in specific regions (Figure 1C). Cumulative GHG emissions from refineries globally

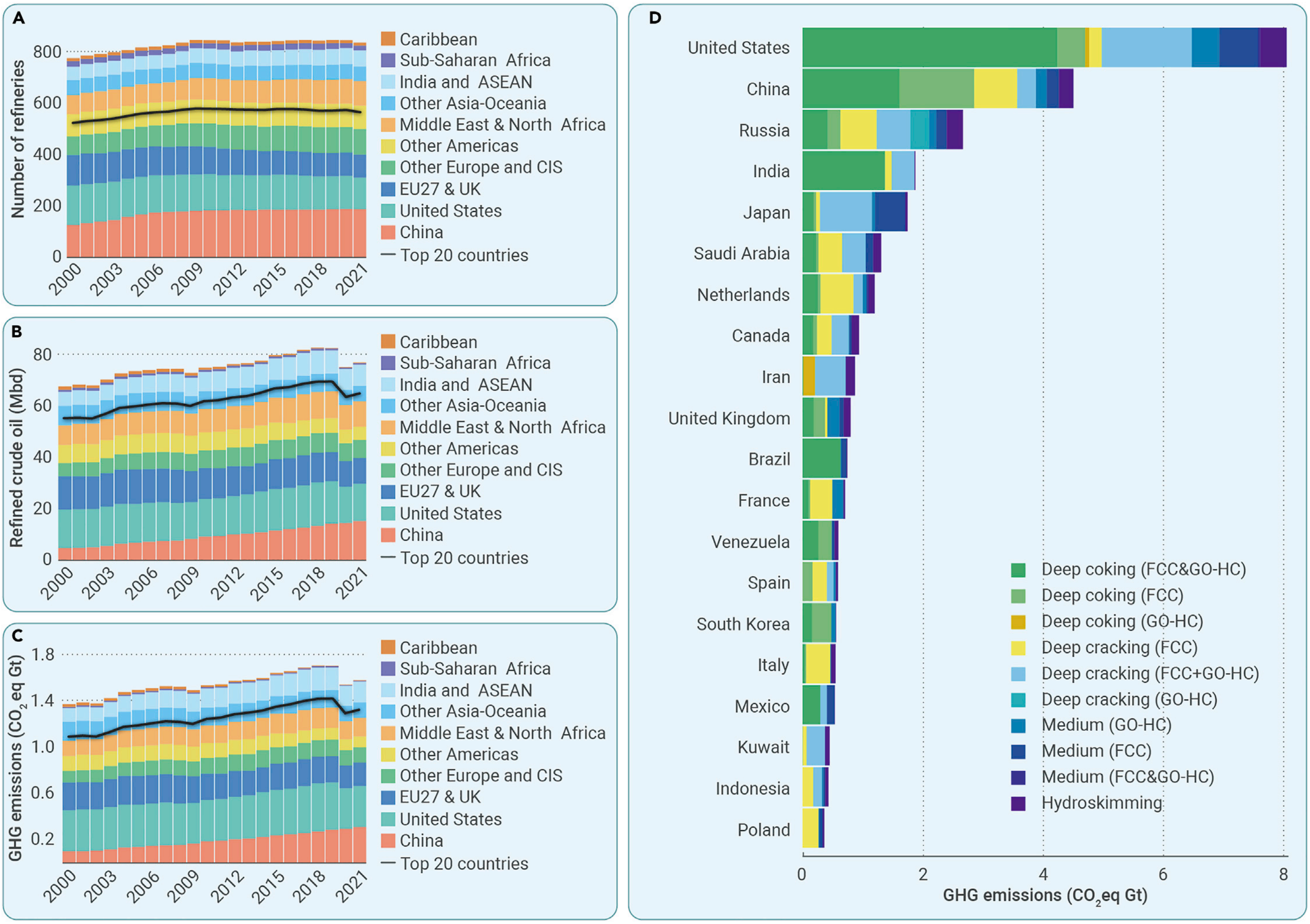

Figure 1. Trends of GHG emissions of global oil refineries from 2000 to 2021 by country, region, and refinery configuration type (A) Changes in number of refineries by regions. (B) Changes in the amount of crude oil refined in the oil refining industry by regions. (C) Trends of GHG emissions by regions. (D) GHG emissions in the top 20 countries with the highest GHG emissions from oil refining industry among all the countries worldwide, which will be named as the top 20 countries later. Note: 10 refinery technology types are included in this study, namely hydroskimming, medium conversion (FCC), medium conversion (GO-HC), medium conversion (FCC&GO-HC), deep coking (FCC), deep coking (GO-HC), deep coking (FCC&GO-HC), deep cracking (FCC), deep cracking (GO-HC), and deep cracking (FCC&GO-HC). Hydroskimming may contain basic process units such as desalter, atmospheric tower furnace, atmospheric tower, naphtha hydrotreater, isomerization unit, etc. In addition to the process units in the hydroskimming refinery, medium conversion (FCC), medium conversion (GO-HC), and medium conversion (FCC & GO-HC) also contains fluid catalytic cracking (FCC) and gas oil hydrocracker (GO-HC). Meanwhile, deep coking (FCC), deep coking (GO-HC), deep coking (FCC&GO-HC), deep cracking (FCC), deep cracking (GO-HC), and deep cracking (FCC&GO-HC) include not only terminal conversion units, but also deep conversion units such as coking or hydrocracking; see details in Table S2. The definition of the 10 regions in this study is shown in Figure S1.

reached approximately 34.1 Gt between 2000 and 2021, with an average annual increasing rate of 0.7%. More than half of the total came from refineries in the United States, EU27&UK, and China, contributing 24.1%, 15.6%, and 12.6%, respectively. From 2000 to 2021, GHG emissions from Chinese refineries more than tripled, from 102.2 to 313.3 Mt, while the country's share of GHG emissions from global oil refining industry has more than doubled from 7.4% to 19.7%. Similar rapid growth was also seen in GHG emissions from India and ASEAN (Association of Southeast Asian Nations) refineries. Meanwhile, the share of EU27&UK refineries' CO_2eq emissions declined from 17.4% to 12.7%, and that of the United States gradually decreased from 25.9% in 2000 to 22.5% in 2021.

Specifically, countries with high GHG emissions usually also show high carbon intensity, which is high GHG emissions from refining a barrel of crude oil. (Figures 1D and S2). GHG emissions from the top 20 countries have become a growing proportion of the total such emissions from the global oil refining sector, accounting for 83.9% of the total in 2021, compared with 79.5% in 2000. The carbon intensity of the top 20 countries is 59.0 kg/bbl, much higher than the world average of 56.2 kg/bbl. Among these countries, the United States, India, Japan, South Korea, Italy, and Spain have relatively high carbon intensities, exceeding 60 kgCO_2eq/bbl. This is because more than 80% of GHG emissions from the oil refining industry of these countries, barring Japan's, came from deep conversion refineries. Countries at the lower end of the top 20 have comparatively low carbon intensity: the United Kingdom, for instance, ranks 15th among these nations and has a carbon emission intensity of just 42.9 kg/bbl.

Trends in the GHG emissions of oil refining enterprises

The 498 enterprises that make up the oil refining industry control 1,095 refineries globally. During 2000–2021, the top 20 enterprises—which comprise just 4% of the 498—dominated global oil refining production and GHG emissions with relatively high carbon intensity (Table S3). Among these top 20 enterprises, five belong to the United States, three to Russia, two to China, and two to India. In addition, Saudi Arabia, Iran, Brazil, the Netherlands, Venezuela, France, the United Kingdom, and Japan each have one enterprise in the top 20. Over the 2000–2021 period, GHG emissions from the top 10 of the 20 and from the remaining enterprises accounted for 33.8%–38.1% and 17.7%–20.5% of the global annual GHG emissions, with their average carbon emission intensity being 61.4–62.4 and 56.3–59.1 kgCO_2eq/bbl, respectively (Figure 2).

Most of the top 20 enterprises came from a few developed countries, or countries with large refining capacities controlled by a local oil refining industry. GHG emissions from oil refining in developing countries are dominated by government-owned enterprises (Table S4). For example, up to 67.0% of GHG emissions in Chinese refineries came from China National Petroleum Corporation and China Petrochemical Corporation. In Iran, the

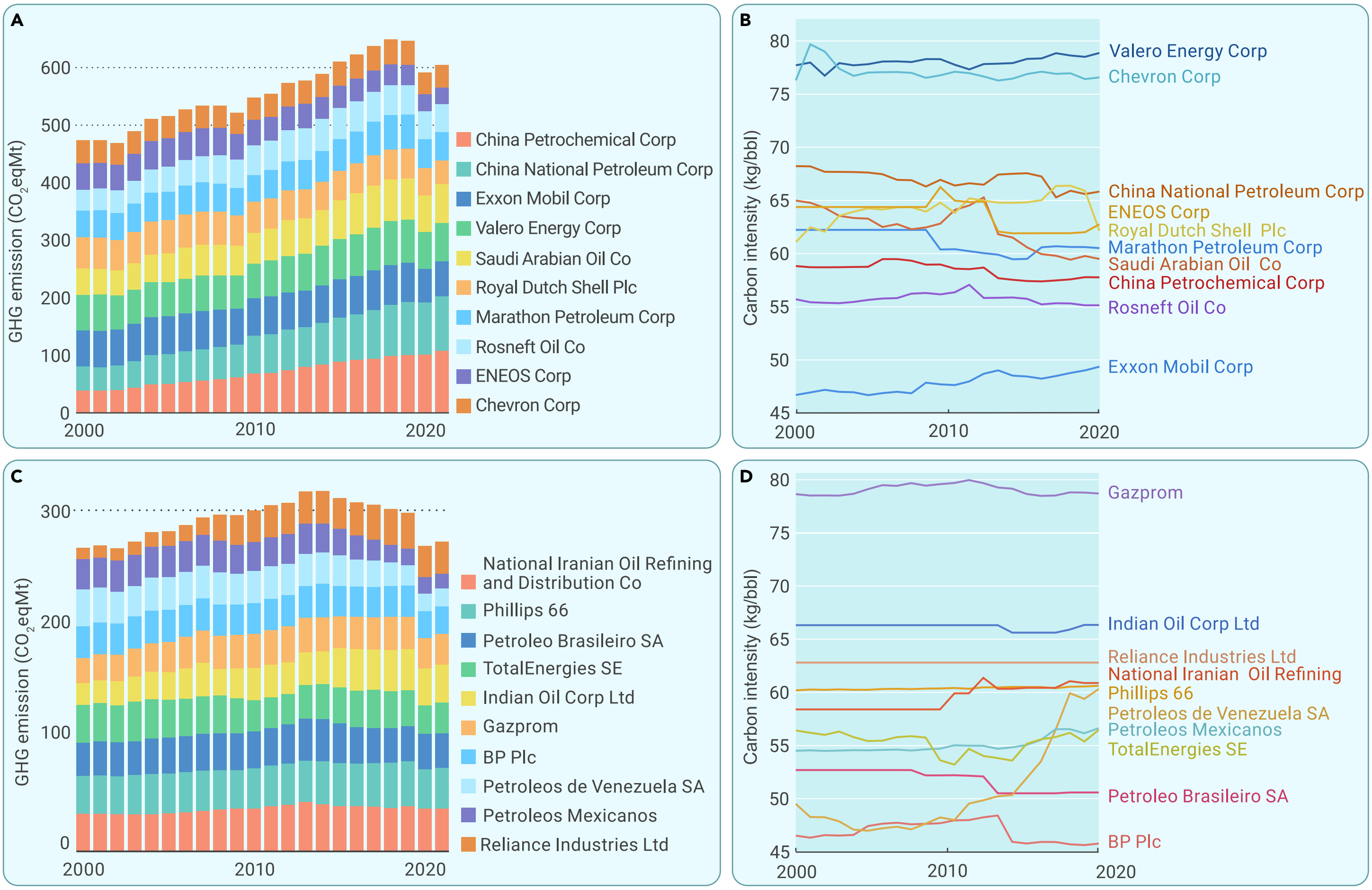

Figure 2. Dynamics in GHG emissions and the carbon intensity of global oil refining enterprises, 2000 to 2021 (A) Trends of GHG emissions in the top 10 enterprises with the highest GHG emissions from the oil refining industry among all the enterprises worldwide, which will be named as the top 10 enterprises later. (B) Trends of carbon intensity in the top 10 oil refining enterprises. (C) Trends of GHG emissions in the top 11–20 enterprises with the highest GHG emissions from oil refining industry among all the enterprises worldwide, which will be named as the top 11–20 enterprises later. (D) Trends of carbon intensity in the top 11–20 oil refining enterprises.

National Iranian Oil Refining and Distribution Company, a government-owned enterprise that essentially controls the country's entire refining industry, is responsible for up to 98.4% of that country's related GHG emissions. BP plc, by contrast, is a special case: an entirely foreign investment enterprise, with all its GHG emissions occurring overseas (Figure S3).

There are three GHG emission patterns for the top 20 enterprises in the last two decades, including stable emissions, rapidly growing emissions, and recessionary emissions. GHG emissions from the oil refining industry in some developed countries show a high and steady trend; cases in point include Valero Energy Corp, Marathon Petroleum Corporation, and Phillips 66 (Figures 2A and 2C), indicating a stable, heavy demand for petroleum products in the United States. Meanwhile, GHG emissions from government-owned oil refining enterprises in emerging countries grew significantly. For example, GHG emissions from the China National Petroleum Corporation and China Petrochemical Corporation had both more than doubled from 2000 to 2019, soaring from 39.2 and 41.7 Mt in 2000 to 100.4 and 92.1 Mt in 2019, respectively. Such an apparent increase also showed in GHG emissions from the Saudi Arabian Oil Company. What drove the boom in GHG emissions from the oil refining industry in these emerging countries was the construction of modern major refineries and the growing domestic demand for refined petroleum products. Conversely, the ENEOS Corporation in Japan declined significantly: its GHG emissions in 2019 were only 78.1% of those in 2000.[20] The carbon intensity of ENEOS Corp also fell by 4% (Figure 2B), indicating shrinkage in the corporation's oil refining capacity, which was caused in turn by a decline in the countries' petroleum demand[21] and by the adjustment of energy structure and energy-saving plans in Japan. A similar sharp decline in GHG emissions could be found in Petroleos de Venezuela SA and Petroleos Mexican OS, from 33.6 to 27.4 Mt in 2000 to 18.6 and 15.2 Mt in 2019, respectively. However, during the same period the carbon intensity of these two enterprises rose by 21.0% and 4.7%, respectively (Figure 2D), due to production cuts and decommissioning of refineries caused by the economic downturn and political turmoil.[22]

Compared with ENEOS Corp, Petroleos de Venezuela SA, and Petroleos Mexican OS, the carbon intensity in most of the top 20 enterprises has remained relatively constant over the past two decades, such as China National Petroleum Corporation and China Petrochemical Corporation. In addition, the top 10 enterprises have higher carbon intensity than the top 11–20 ones: except for Exxon Mobil Corporation, the average carbon intensity of those top 10 is higher than 55 kg CO_2eq/bbl, while only half of the top 11–20 enterprises have an average carbon intensity higher than 55 kg CO_2eq/bbl (Figures 2B and 2D). This could mean that enterprises with high GHG emissions also have high carbon intensity.

GHG emissions in the oil refining processes

Currently, the installation of abatement facilities is focused on four main processes in refining: electricity, furnaces and boilers, fluid catalytic cracking (FCC), and steam methane reforming (SMR).[23] We integrated the remaining process emissions such as support services emissions and releasing from managed wastes into other emissions. Therefore, based on the life cycle method and the existing crude oil sample database, we estimated and summarized the GHG emissions of five process units in 10 refinery configurations, as shown in Figure 3A. GHG emissions from furnaces and boilers and electricity in hydroskimming refineries account for 66.2% and 26.5%, respectively, of total GHG emissions. However, in medium conversion refineries, FCC or gas oil hydrocracking (GO-HC) contributes a considerable amount of GHG emissions, so these have emerged as the second-largest

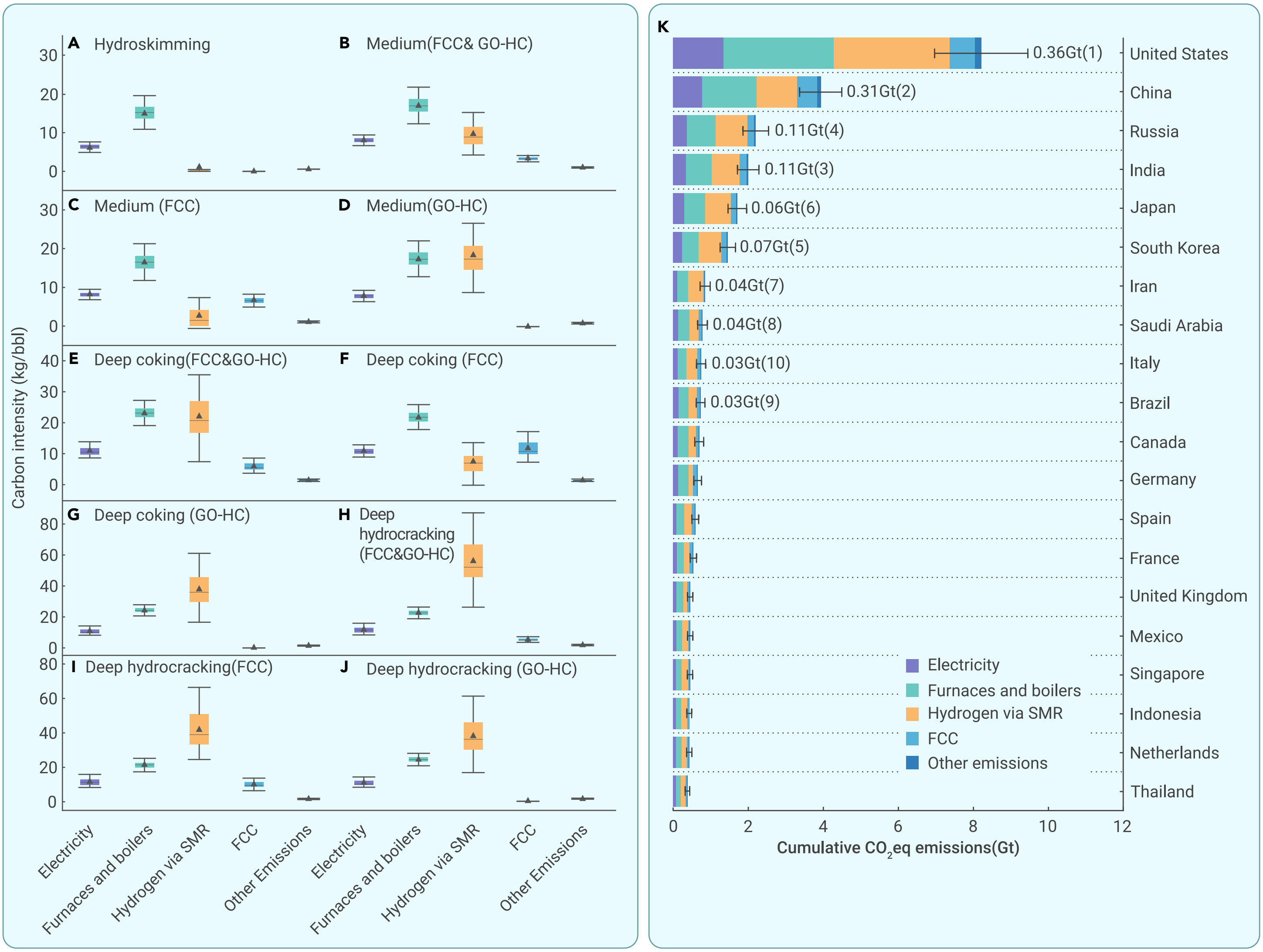

Figure 3. GHG emissions based on process units GHG emissions based on process units in (A) hydroskimming refineries, (B) medium conversion refineries (FCC), (C) medium conversion refineries (GO-HC), (D) medium conversion refineries (FCC&GO-HC), (E) Deep coking refineries (FCC), (F) deep coking refineries (GO-HC), (G) deep coking refineries (FCC&GO-HC), (H) deep hydrocracking refineries (FCC), (I) deep hydrocracking refineries (GO-HC), and (J) deep hydrocracking refineries (FCC&GO-HC). (K) Cumulative GHG emissions based on process units in the top 20 countries. The number in Figure 2K represents GHG emissions from the refining industry in the top 20 countries in 2021.

GHG emission process in refineries (Figures 3B–3D). Compared with hydroskimming refineries, the proportion of GHG emissions generated by furnaces and boilers is relatively small, accounting for 40.3%, 32.5%, and 36.3% in deep coking refineries (FCC), deep coking refineries (GO-HC), and deep coking refineries (FCC&GO-HC), respectively (Figures 3E–3G). Hydrogen via SMR is the largest GHG emitter in deep hydrocracking refineries, accounting for 48.1%, 66.4%, and 58.0% in deep hydrocracking refineries (FCC), deep hydrocracking refineries (GO-HC), and deep hydrocracking refineries (FCC&GO-HC), respectively (Figures 3H–3J).

In the top 20 countries, GHGs from furnaces and boilers accounted for 30.1%–43.5% of total cumulative refinery GHG emissions from 2000 to 2021 (Figure 3K), while this proportion increased to 47.0%–67.2% in the 20 countries with the lowest emissions from the refining sector (Figure S4). This is mainly due to the relatively high proportion of deep conversion refineries that have relatively low GHG emissions from furnaces and boilers in the top 20 countries. For example, deep conversion refineries in the United States accounted for 81.1% of total refinery production in 2021, resulting in 35.8% of GHG emissions from heating processes, while there was all simple hydroskimming refineries in Laos leading to 65% of GHG emissions from heating processes (Figure S5). Hydrogen via SMR was also a crucial GHG emission processes in oil refineries in top 20 countries, accounting for 17.7%–49.2% (Figure 3). This is because medium and heavy conversion refineries require large amounts of hydrogen to convert heavy oil into light oil. Moreover, electricity was the third-largest source of GHG emissions from refineries, contributing 13.1%–20.7% of the total emissions in the top 20 countries.

The process emissions in the top refineries controlled by the enterprise with the largest GHG emissions in each region were analyzed (Figure 4). Notably, there are more than 20% of their refineries with GHG emissions exceeding 0.1 Gt in five enterprises including Valero Energy Corporation, Exxon Mobil Corporation, Royal Dutch Shell Plc, Saudi Arabian Oil Company, and Indian Oil Corporation Ltd. The GHG emissions of these refineries accounted for 55.8%–84.0% of total such emissions in these five enterprises, respectively. The top 20% refineries with the largest GHG emissions accounted for 38.0%–59.5% of total GHG emissions in another six enterprises including China Petrochemical Corporation, China National Petroleum Corporation, Marathon Petroleum Corporation, Resneft Company, Petroleum Brasilero SA, and ENEOS Corporation. We further analyzed the GHG emissions of refining processing units within the top refineries controlled by these enterprises, within the context of reducing emissions (Figure 4B). The four main emissions sources associated with processing—electricity, furnaces and boilers, hydrogen via SMR and FCC—accounted for 96.9%–98.8% of total GHG emissions. However, due to the difference in configuration types of refineries, low-carbon deployment pathways for each refinery are not straightforward. For example, hydrogen via SMR is the largest GHG emission source in the Fujian refinery, while heat and steam constitute the largest in the Maoming refinery. Plants will inevitably need to factor the type of source into their mitigation strategies, for instance in prioritizing which refining units must

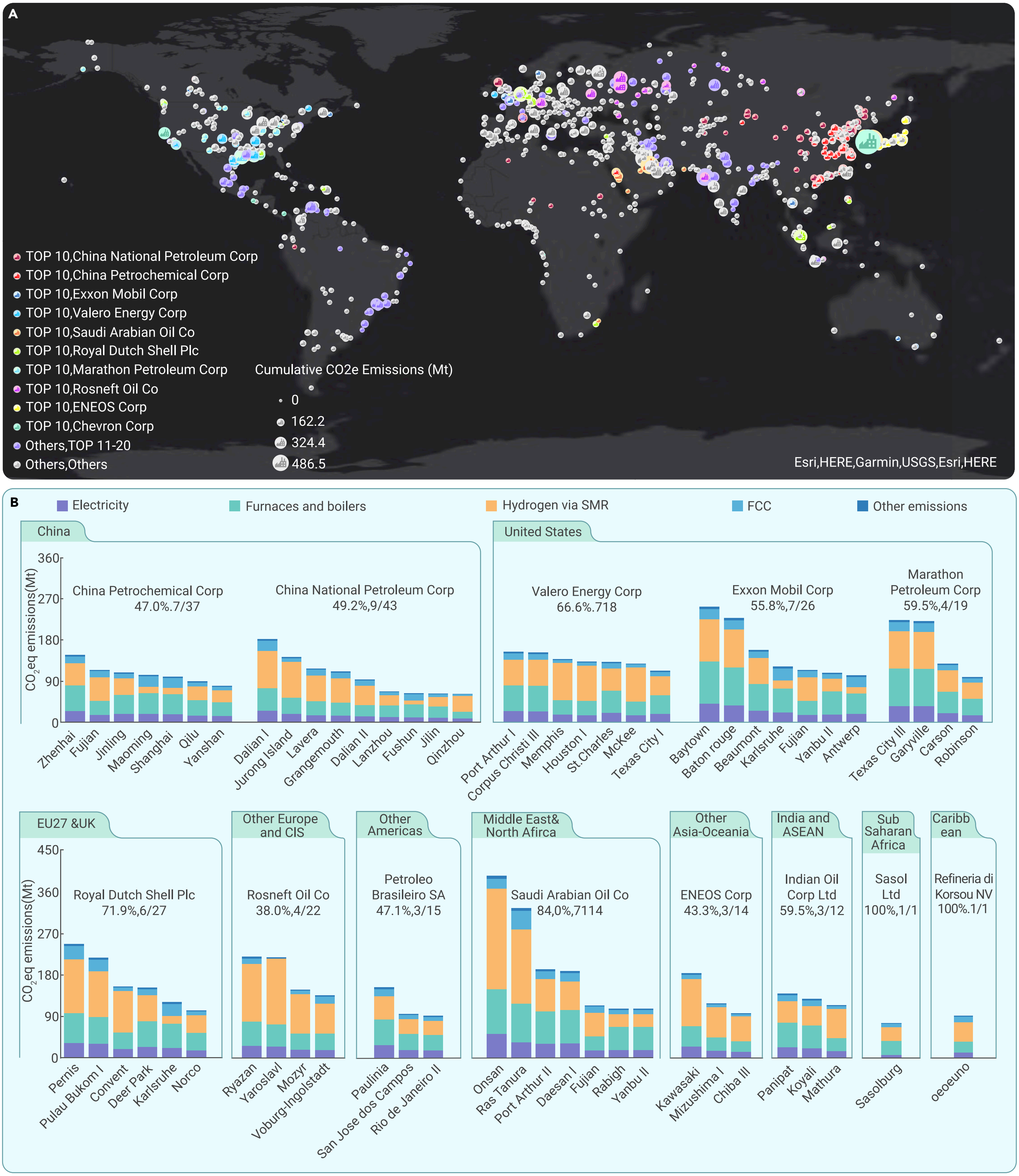

Figure 4. Cumulative process GHG emissions of crucial enterprises from 2000 to 2021 (A) Location, enterprises, and cumulative GHG emissions of 1,405 oil refineries worldwide. Color of the point shows the refining enterprises to which the refinery belongs. The size of points indicates the cumulative GHG emissions size (≤162.2 Mt, ≤324.4 Mt, ≤486.5 Mt). (B) The process GHG emissions of top refineries in the enterprise with the largest GHG emissions in each region. The notes in Figure 4B respectively show the proportion of GHG emissions of important refineries in the enterprise and the proportion of key refineries in the total refineries in the enterprise. The top 20% refineries with the largest GHG emissions in an enterprise or refineries with GHG emissions more than 0.1 Gt controlled by an enterprise were named as top refineries. The process GHG emissions of top refineries in the other top 20 enterprises are shown in Figure S6.

install GHG mitigation technologies. A refinery GHG emission inventory based on the life cycle method can thus provide basic data support for the deployment of each refinery emission reduction technology.

DISCUSSION

This study built CEADs-GREI v2.0 as a global sub-refinery GHG emission database based on the life cycle method. This expands on an earlier version, CEADs-GREI,[10] by adding and exploring GHG emissions from the sub-refinery level. Moreover, it identifies three kinds of major contributors to refinery GHG emissions: highest-emitting countries to reduce GHG emissions, enterprises with crucial responsibilities, and key refinery processes, thus providing more detailed basic data support for targeted GHG emissions reduction in the oil refining industry. We estimated the current global GHG emissions from refineries in successive years, which will depend on the type and output of refineries, and we did not consider the implementation of mitigation strategies in the oil refining sector. Previous studies on GHG emissions from global oil refineries based on point sources focused on the comparison of carbon intensity and GHG emissions among countries.[9,10] This study is the first to examine national process unit-based GHG emissions of the oil refining industry from a sub-refinery scale, highlighting the important contribution of key process units in a few countries to GHG emissions from global oil refineries. Moreover, we also highlighted the importance of a small number of major enterprises and oil refineries in global GHG emissions from the oil refining industry.

Key mitigation strategies for countries and enterprises

The top 20 countries with the highest GHG emissions from the global oil refining industry are at the core of GHG emission reduction in each region (Table S5), while the 20 enterprises with the highest GHG emissions are located in these 20 countries, which play an important role in the GHG emissions of the refining industry in these countries (Figure 2 and Table S3). GHG emissions from refineries in energy-leading developed countries like the United States have been both huge and stable. These countries should take proactive measures to reduce GHG emissions in the oil refining sector, such as energy transformation to achieve refinery production reduction or decommissioning or the deployment of carbon capture, utilization, and storage (CCUS).[8,11] Another key point is that the largest enterprises with the largest GHG emissions play a significant role in GHG emissions from refineries in such countries and are privately owned. For example, five of the top 20 enterprises in the United States, Valero Energy, Phillips 66, Marathon Petroleum, ExxonMobil, and Chevron, are all non-government-owned (Table S4), yet they accounted for 53.6% of GHG emissions from the US oil refining industry as a whole. Elements of the free market economic system make it difficult for the government to control these enterprises strictly.[18] Thus effective emission reductions in this sector in such countries depends on the independent actions of the companies themselves.

In some developed countries, including the United Kingdom and Japan, the demand for petroleum products is declining even as an energy transformation is being actively promoted. These nations are likely to continue reducing GHG emissions from the oil refining industry in the future.[10] Privately owned oil refining companies also dominated GHG emissions in these countries. Given the industry's downturn in them, there may be a greater need for state financial subsidies to encourage companies to install mitigation facilities for their refineries in these countries. By contrast, emerging countries like China and India need an increasing volume of fossil fuels such as oil products to support their economic growth, thus increasing the number of refineries and expanding their refinery capacity to meet demand (Figures 1A and 1B). As a result, these countries become the main driver of increased GHG emissions from the global refining industry.[24,25] These countries should install emission-reduction facilities in their refineries and take specific low-carbon actions such as embarking on an energy transformation to control demand for refinery products while also meeting the climate target. Government-owned enterprises, including the China National Petroleum Corporation, China Petrochemical Corporation, and Indian Oil Corporation Ltd, dominated national GHG emissions from refineries in these nations.[26] Government policy intervention may thus push them to achieve effective mitigation measures. GHG emissions from the refining industry in some developing countries such as Venezuela, however, have been declining due to underinvestment caused by the financial crisis and political and domestic turmoil.[22] It is thus difficult to demand that they reduce GHG emissions in the near term.

National and corporate GHG emission reduction will eventually be implemented through the installation of mitigation facilities in crucial refineries. The carbon intensity of most of the top 20 enterprises remained stable or varied slightly over time, mainly because the new construction and decommissioning of a few refineries did not lead to drastic changes in the refinery configuration structure of these enterprises. Thus, refineries with rates of GHG emissions in the top 20% and those with GHG emissions greater than 0.1 Gt have consistently dominated the change in carbon intensity of these enterprises and have accounted for 54.0% of GHG emissions of the top 20 enterprises over the past two decades. The deployment of abatement facilities in these refineries will significantly reduce GHG emissions from the refining industry in the top 20 companies. For example, achieving net-zero carbon emissions from seven refineries with largest carbon emissions in China Petrochemical Corporation would reduce its GHG emissions by 47% (Figure 4). Moreover, our results show that furnaces and boilers as well as hydrogen via SMR are process units with the highest GHG emissions at the refinery, accounting for at least 54.0% of total refinery emissions, similar to findings in previous studies.[27,28] Prioritizing the installation of carbon mitigation technologies according to the configuration of the refinery's processing units is essential if each refinery is to achieve GHG reductions. Furthermore, the significant disparity in the CO_2 content of exhaust gas and the aggregation of the same process unit in refineries will further affect the difficulty and cost of implementing CO_2 capture and storage (CCS) in these processes.[29] For example, due to the ubiquity of furnaces and boilers in refineries causing the difficulty of collecting GHG in these units, post-combustion CCS at these unit will be limited; exhaust gas generated by hydrogen production, however, has high CO_2 purity (20%–99%), which could be easier to capture at a lower cost.[23] Therefore, compared with the top 11–20 countries, it is easier for the top 10 countries to reduce GHG emissions at a lower economic cost early on because of the small proportion of GHG emissions from furnaces and boilers. Analyzing GHG emissions during the refining process in key refineries will support the targeted implementation of emission reduction technologies, and thus drive countries and enterprises to take action on mitigation.[9,30]

The uncertainty of estimating GHG emissions from global refineries

The uncertainty associated with estimating GHG emissions from refineries is mainly caused by the type of refinery configuration, the type of crude oil, and so on.[31] We assigned 148 crude oil samples to three types of refineries based on the rules laid out in Table S6. However, this rough rule brought great uncertainty to the estimation of GHG emissions from refineries with different configurations in our study. The uncertainty of estimating GHG emissions from the global oil refining industry is shown in Figure S7. The GHG emissions from the global refining industry estimated in this study increased from 1.38 ± 0.21 Gt in 2000 to 1.59 ± 0.24 Gt in 2021. To test the reliability of the data, we also compared our results with previous studies. Jing et al.[9] and Lei et al.[10] reported global refining sector GHG emissions of 1.2 and 1.3 Gt in 2015 and 2018, respectively, while the GHG emissions calculated in this study in the same years were 38% and 32% higher than those in previous studies (Table S7). This may be because there is a wider range of data, covering 1,195 refineries in operation in 121 countries in the CEADS-GREI v2.0 database.

The demand for a near-real-time refinery GHG emission database

In the future, we hope to be able to monitor the GHG emission reduction of global refineries in near real time in CEADS-GREI v3.0, with more refined timescale GHG emissions at the month level and smaller time lag of GHG emission report from oil refineries worldwide. A near-real-time refinery GHG emission database could also supply detailed, accurate data support for the oil refining industry to achieve energy transition and GHG emission reduction targets, especially in the wake of major natural and social disasters, such as COVID-19, the 2008 economic meltdown, and so on.[32] Meanwhile, we plan to collect information on mitigation technologies that have

been installed or have been planned to be installed in global refineries before 2060 to monitor and supervise the realization of GHG emission reduction targets worldwide.

MATERIALS AND METHODS

New CEADs-GREI

On the basis of the original database, we integrated multiple datasets and established the CEADs-GREI v2.0, whose data accuracy reached the sub-refinery level for the first time.[10] (See Table S1 for data sources and basic database information.) Compared with the original database, the new version 2.0 mainly refined three parameters: (1) ownership structure of each refinery (including the top seven shareholders and their shares); (2) all industrial processes of the refinery (including process units, service life, and crude oil refining capacity of process units for successive years); and (3) three refinery configuration types, refined into 10 refinery configuration types. The crude oil refining amount (CORA) of consecutive years and months is obtained through standardization:

We estimated the CORA of global refineries from January 2000 to June 2021 using the following equation.

$$P_{m,n,Y} = CA_{m,n,Y}/CA_{n,Y} * P_{n,Y} * 1000 * 365, \qquad \text{(Equation 1)}$$

where $P_{m,n,Y}$ represents the CORA of refinery m of country n in year Y, bbl; $CA_{m,n,Y}$ represents daily crude oil refining capacity (CORC) of refinery m of country n in year Y, thousands of barrels per day (kbd); $CA_{n,Y}$ represents daily CORC of country n in year Y, kbd; $P_{n,Y}$ represents daily CORA of country n in year Y, kbd. $CA_{m,n,Y}$ and $CA_{n,Y}$ come from CEADs-GREI database: https://www.ceads.net.cn/. $P_{n,Y}$ comes from the BP Statistical Review of World Energy 2021.[33]

Construction of GHG emissions database based on refinery industrial process unit

This study used PRELIM version 1.5. PRELIM is the first open-source tool capable of estimating GHG emissions and energy use in different refinery types, which can be allocated to all process units by using the life cycle method. The system boundary of the PRELIM model is the boundary of the refinery itself, including all major process units as well as all energy and hydrogen supplied to the refinery.[9] The functional unit in PRELIM is 1 bbl input crude oil. PRELIM can simulate 10 refinery configurations based on the complexity of refining and how they deal with heavy fractions of crude oil. The model details of this model could be found in Bergerson et al. (2020).[34] Therefore, this study can break refinery configurations down into 10 types, compared with four types in the CEADs-GREI v1.0.[10] Detailed characteristics of the ten configurations of refineries are shown in Table S2. Details of basic background parameters set of PRELIM model in this study can be found in Table S8. PRELIM version 1.5 contains detailed parameters of 148 crude oil samples available in various regions of the world.

Based on the database, we used the PRELIM model to calculate the life cycle GHG emissions of each oil sample under different refinery configurations, and we used the average to estimate the total GHG emissions of each refinery configuration and each industrial process unit, with standard deviation representing their uncertainty (Equations 2 and 3).[35]

$$Cmean_{b,c} = \sum_{a=1}^{n} C_{a,b,c}/n \qquad \text{(Equation 2)}$$

$$Csd_{b,c} = \sqrt{\sum_{a=1}^{n} \left(C_{a,b,c} - Cmean_{b,c}\right)^2}, \qquad \text{(Equation 3)}$$

where $Cmean_{b,c}$ represents the average GHG emissions of 1 kg crude oil sample of the process c in the refinery of type b, kg/bbl; $C_{a,b,c}$ represents GHG emissions of 1 kg crude oil sample a of the process c in the refinery of type b, kg/bbl. b Includes 10 refinery types; c contains five kinds of industrial process unit, including heaters and boilers, hydrogen production via SMR, utilities, FCC, and subprocesses. $Csd_{b,c}$ represents the standard deviation of GHG emissions of 1 kg crude oil sample in process c in the refinery of type b, kg/bbl.

GHG emissions accounting

This study analyzed sub-refinery GHG emissions from three aspects (industrial process, more refined timescale, and ownership structure). The total GHG emissions per unit of crude oil and per industrial process in different refinery configurations were obtained by using the PRELIM model. The CORA of a single refinery in consecutive years was obtained through standardization and dimension reduction. Therefore, we estimated annual GHG emissions and industrial process GHG emissions using Equation 4. The GHG emissions of holding companies were obtained by splitting and summarizing the GHG emissions of a single refinery in proportion. The ownership structure we used is the data of the enterprises in 2021.

$$CE_{m,n,Y} = \sum_{c=1}^{5} Cmean_{b,c} * P_{m,n,Y}, \qquad \text{(Equation 4)}$$

where $CE_{m,n,Y}$ represents refinery GHG emissions of refinery m of country n in year Y, kg, which is calculated at sub-refinery scale.

REFERENCES

1. Bliss, A.C., Steele, M., Peng, G., et al. (2019). Regional variability of Arctic sea ice seasonal change climate indicators from a passive microwave climate data record. Environ. Res. Lett. **14**, 045003.
2. van Soest, H.L., den Elzen, M.G.J., and van Vuuren, D.P. (2021). Net-zero emission targets for major emitting countries consistent with the Paris Agreement. Nat. Commun. **12**, 2140.
3. Tong, D., Zhang, Q., Zheng, Y., et al. (2019). Committed emissions from existing energy infrastructure jeopardize 1.5°C climate target. Nature **572**, 373–377.
4. IPCC (2018). Global Warming of 1.5°C. In An IPCC Special Report on the Impacts of Global Warming of 1.5°C above Pre-industrial Levels and Related Global Greenhouse Gas Emission Pathways, in the Context of Strengthening the Global Response to the Threat of Climate Change, Sustainable Development, and Efforts to Eradicate Poverty (Cambridge University Press).
5. Wang, P., Ryberg, M., Yang, Y., et al. (2021). Efficiency stagnation in global steel production urges joint supply- and demand-side mitigation efforts. Nat. Commun. **12**, 2066.
6. IPCC (2021). Summary for policymakers. In Climate Change 2021: The Physical Science Basis. Contribution of Working Group I to the Sixth Assessment Report of the Intergovernmental Panel on Climate Change (Cambridge University Press).
7. Mi, Z., Zheng, J., Meng, J., et al. (2019). Carbon emissions of cities from a consumption-based perspective. Appl. Energy **235**, 509–518.
8. IEA (2021). World Energy Outlook 2021 (International Energy Agency).
9. Jing, L., El-Houjeiri, H.M., Monfort, J.-C., et al. (2020). Carbon intensity of global crude oil refining and mitigation potential. Nat. Clim. Chang. **10**, 526–532.
10. Lei, T., Guan, D., Shan, Y., et al. (2021). Adaptive CO_2 emissions mitigation strategies of global oil refineries in all age groups. One Earth **4**, 1114–1126.
11. IEA (2020). Energy Technology Perspectives 2020 (International Energy Agency).
12. Walls, W. (2010). Petroleum refining industry in China. Energy Pol. **38**, 2110–2115.
13. Powell, J.T., Pons, J.C., and Chertow, M. (2016). Waste informatics: establishing characteristics of contemporary US landfill quantities and practices. Environ. Sci. Technol. **50**, 10877–10884.
14. Shan, Y., Guan, D., Zheng, H., et al. (2018). China CO_2 emission accounts 1997–2015. Sci. Data **5**, 170201.
15. US EPA (2017). Greenhouse gas reporting program (GHGRP), U.S. Environmental Protection Agency. https://www.epa.gov/ghgreporting.
16. IFP Energies Nouvelles. (2016). Overview of the Refining Industry in the European Union Emissions Trading System.
17. IEA (2020). The Oil and Gas Industry in Energy Transitions (International Energy Agency).
18. Dietz, S., Gardiner, D., Jahn, V., et al. (2021). How ambitious are oil and gas companies' climate goals? Science **374**, 405–408.
19. Motazedi, K., Abella, J.P., and Bergerson, J.A. (2017). Techno-economic evaluation of technologies to mitigate greenhouse gas emissions at North American refineries. Environ. Sci. Technol. **51**, 1918–1928.
20. Emily, B., and Tim, F. (2019). Global refining: profitting in a downstream downturn. https://www.mckinsey.com/industries/oil-and-gas/our-insights/global-refining-profiting-in-a-downstream-downturn.
21. PAJ (2020). Petroleum Industry in Japan (Petroleum Association of Japan).
22. Monaldi, F. (2015). The Impact of the Decline in Oil Prices on the Economics, Politics and Oil Industry of Venezuela (Columbia Center on Global Energy Policy).
23. IEA/UNIDO (2011). Technology Roadmap Carbon Capture and Storage in Industrial Applications (International Energy Agency and United Nations Industrial Development Organization).
24. Zaman, K., and Moemen, M.A.E. (2017). Energy consumption, carbon dioxide emissions and economic development: evaluating alternative and plausible environmental hypothesis for sustainable growth. Renew. Sustain. Energy Rev. **74**, 1119–1130.
25. Malik, A., Lan, J., and Lenzen, M. (2016). Trends in global greenhouse gas emissions from 1990 to 2010. Environ. Sci. Technol. **50**, 4722–4730.
26. Herr, H. (2018). Underdevelopment and Unregulated Markets: Seven Reasons Why Unregulated Markets Reproduce Underdevelopment (Berlin School of Economics and Law, Institute for International Political Economy (IPE)).
27. Bains, P., Psarras, P., and Wilcox, J. (2017). CO_2 capture from the industry sector. Prog. Energy Combust. Sci. **63**, 146–172.
28. Elgowainy, A., Han, J., Cai, H., et al. (2014). Energy efficiency and greenhouse gas emission intensity of petroleum products at US refineries. Environ. Sci. Technol. **48**, 7612–7624.
29. Veritas, D.N. (2011). Global Technology Roadmap for CCS in Industry Sectoral Assessment: Refineries (United Nations Industrial Development Organization).
30. Klaaßen, L., and Stoll, C. (2021). Harmonizing corporate carbon footprints. Nat. Commun. **12**, 6149.

31. Berghout, N., Meerman, H., van den Broek, M., et al. (2019). Assessing deployment pathways for greenhouse gas emissions reductions in an industrial plant - a case study for a complex oil refinery. Appl. Energy ***236***, 354–378.
32. Urban, F., Mitchell, T., and Villanueva, P.S. (2011). Issues at the interface of disaster risk management and low-carbon development. Clim. Dev. ***3***, 259–279.
33. BP (2021). Statistical Review of World Energy, 70th edition (British Petroleum).
34. Bergerson, J.A., Abella, J.P., Motazedi, K., et al. (2020). Petroleum refinery life cycle inventory model (PRELIM). https://ucalgary.ca/sites/default/files/teams/477/PRELIM-v1.5-Documentation.pdf.
35. van der Spek, M., Fout, T., Garcia, M., et al. (2020). Uncertainty analysis in the techno-economic assessment of CO_2 capture and storage technologies. Critical review and guidelines for use. Int. J. Greenhouse Gas Control ***100***, 103113.

ACKNOWLEDGMENTS

We acknowledge supports from the National Key R&D Program of China (2022YFE0208500), the National Natural Science Foundation of China (41921005 and 72140001) and the UK Natural Environment Research Council (NE/V002414/1 and 2021GRIP02COP-AQ), the Royal Society (IEC/NSFC/191520).

AUTHOR CONTRIBUTIONS

D.G. and J.M. designed the study. S.M. performed the analysis. S.M. and T.L. prepared the manuscript. All authors (S.M., T.L., J.M., X.L., and D.G.) participated in the writing of the manuscript.

DECLARATION OF INTERESTS

The authors declare no competing interests.

SUPPLEMENTAL INFORMATION

It can be found online at https://doi.org/10.1016/j.xinn.2022.100361.

LEAD CONTACT WEBSITE

Jing Meng: https://www.ucl.ac.uk/bartlett/construction/people/dr-jing-meng.
Dabo Guan: http://faculty.dess.tsinghua.edu.cn/guandabo/zh_CN/index.htm.

The Innovation, Volume *4*

Supplemental Information

Contributions of key countries, enterprises, and refineries to greenhouse gas emissions in global oil refining, 2000–2021

Shijun Ma, Tianyang Lei, Jing Meng, Xi Liang, and Dabo Guan

Supplemental information for
The contributions of key countries, enterprises and refineries to greenhouse gas emissions in global oil refining 2000-2021

Ma et al.

Supplemental Figures 3
Figure S1 Definition of ten regions in the world 3
Figure S2 GHG emissions in the top 20 countries with the highest GHG emissions from oil refining industry among all the countries worldwide 4
Figure S3 Domestic and overseas GHG emissions of the top 20 enterprises 5
Figure S4 GHG emission composition of process units in the 20 countries with the lowest GHG emissions from the refining industry. 6
Figure S5 Composition of refinery production in the top 20 countries and the bottom 20 countries for GHG emissions from the refining industry 7
Figure S6 GHG emissions of important refineries by refining process in other top 20 enterprises 9
Figure S7 Uncertainty analysis of GHG emissions from global refineries from 2000 to 2021 10
Supplemental Tables 11
Table S1 Data sources and details of CEADs-GREIv2.0 11
Table S2 Details of the possible existing process units in each configuration. 12
Table S3 Cumulative GHG emissions of oil refining enterprises from 2000 to 2021 14
Table S4 Detailed information of top 20 countries 32
Table S5 Cumulative GHG emissions of top 20 countries 33
Table S6 Crude oil classification based on API gravity and sulfur content[1] 35
Table S7 Comparison between the global refining industry GHG emissions estimated by this work and previous studies 36
Table S8 Default Settings for the PRELIM model 37
Reference 37

Supplemental Figures

Figure S1 Definition of ten regions in the world

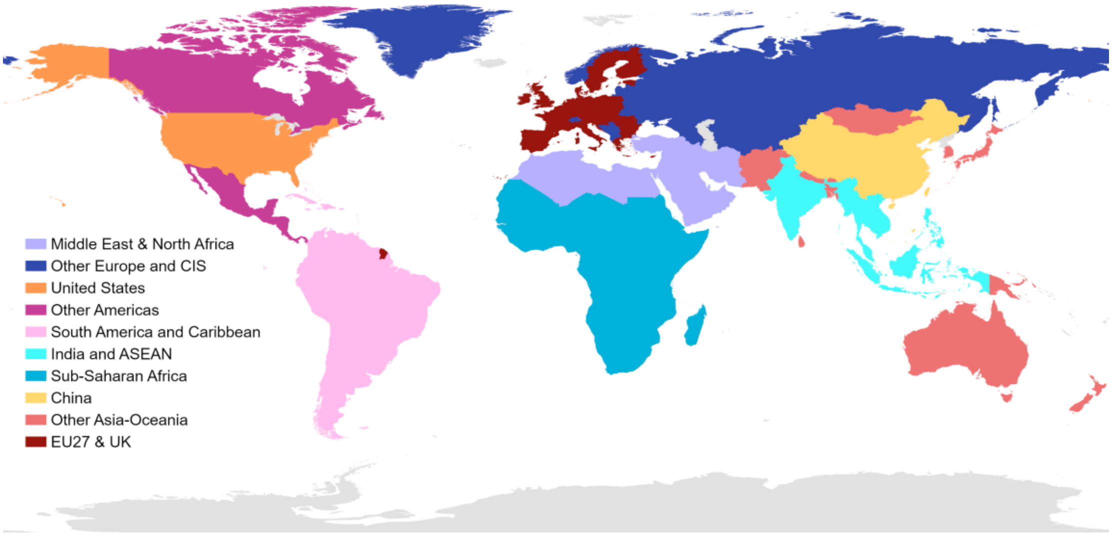

Figure S2 GHG emissions in the top 20 countries with the highest GHG emissions from oil refining industry among all the countries worldwide

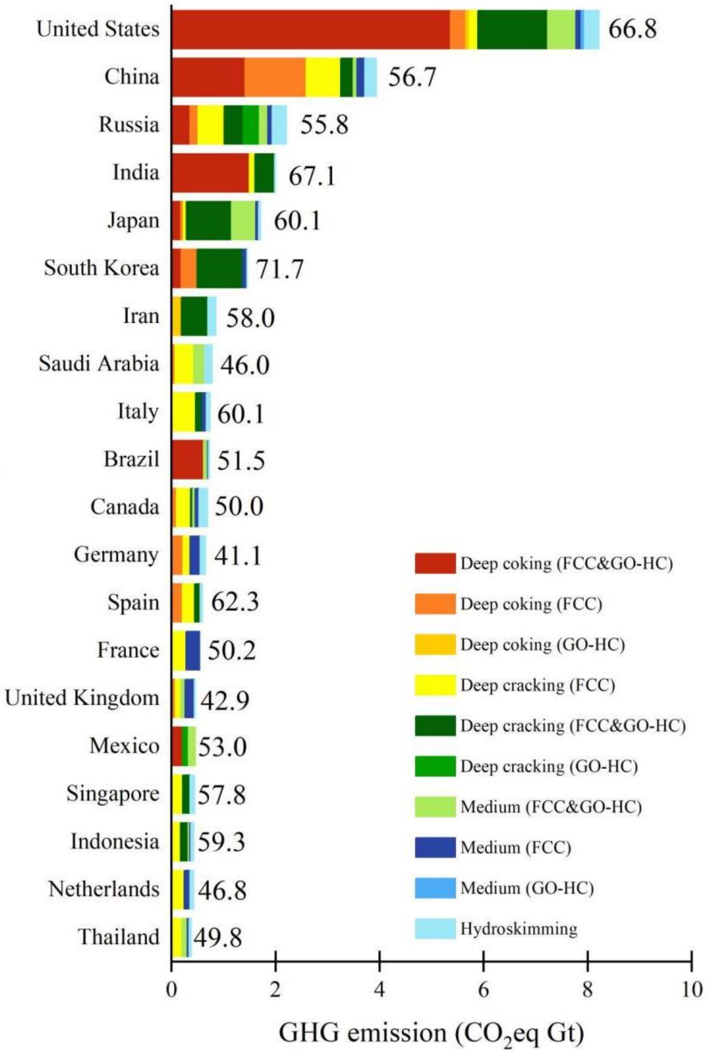

Figure S3 Domestic and overseas GHG emissions of the top 20 enterprises

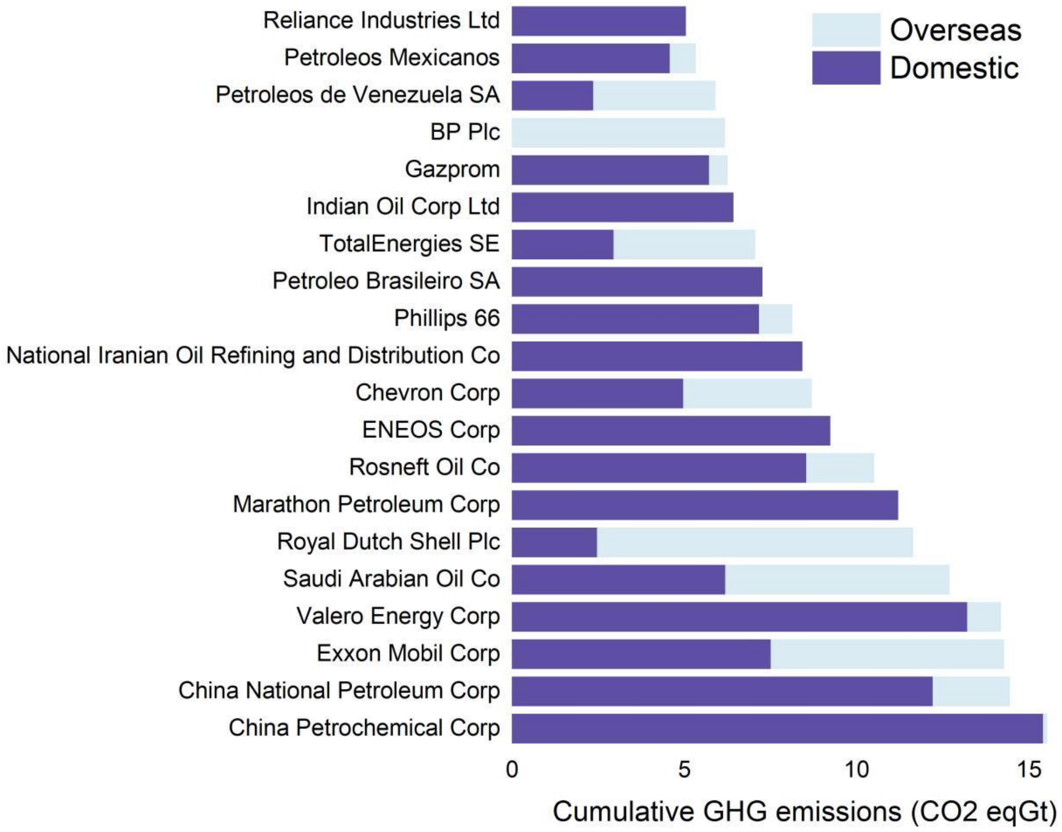

Figure S4 GHG emission composition of process units in the 20 countries with the lowest GHG emissions from the refining industry.

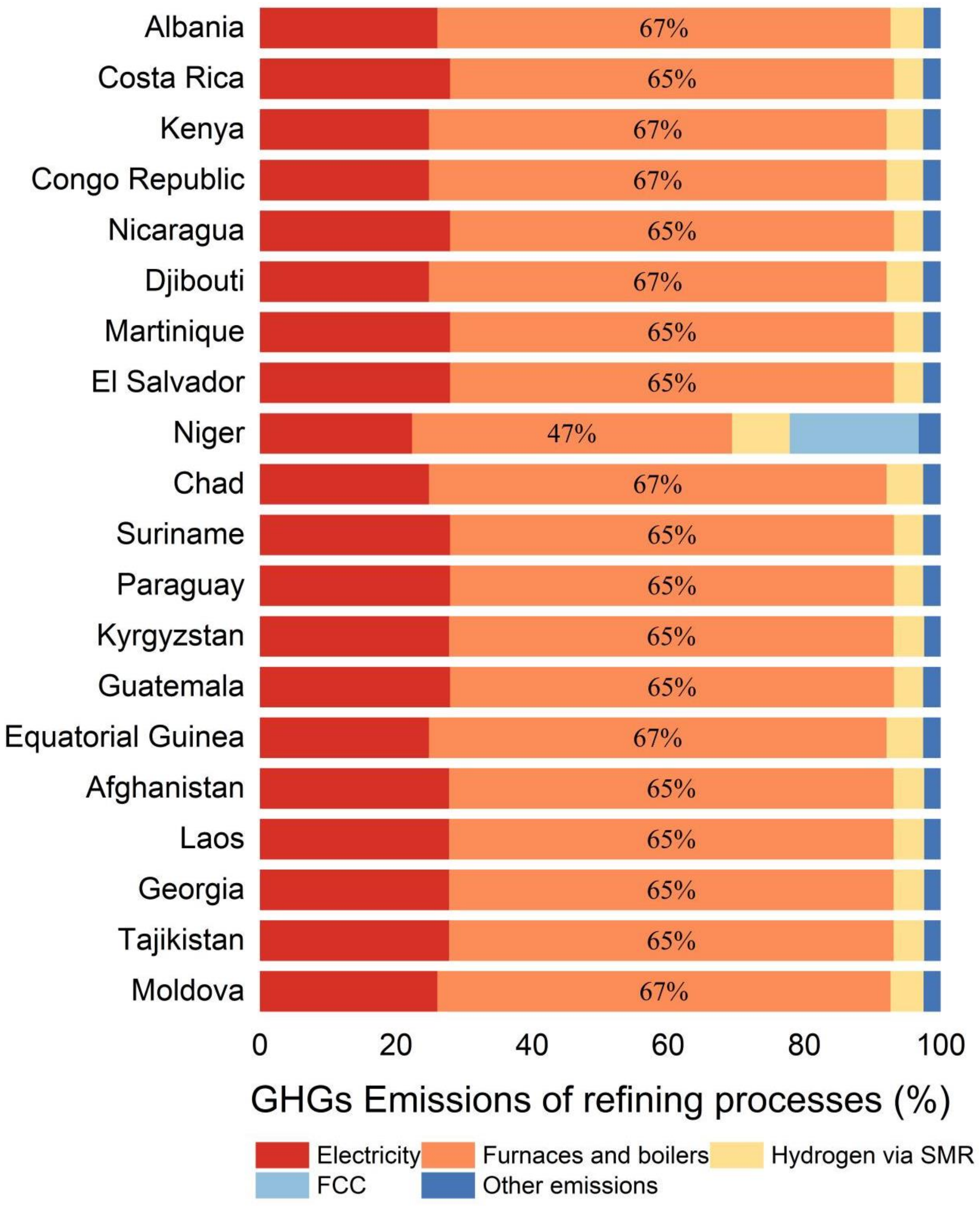

Figure S5 Composition of refinery production in the top 20 countries and the bottom 20 countries for GHG emissions from the refining industry

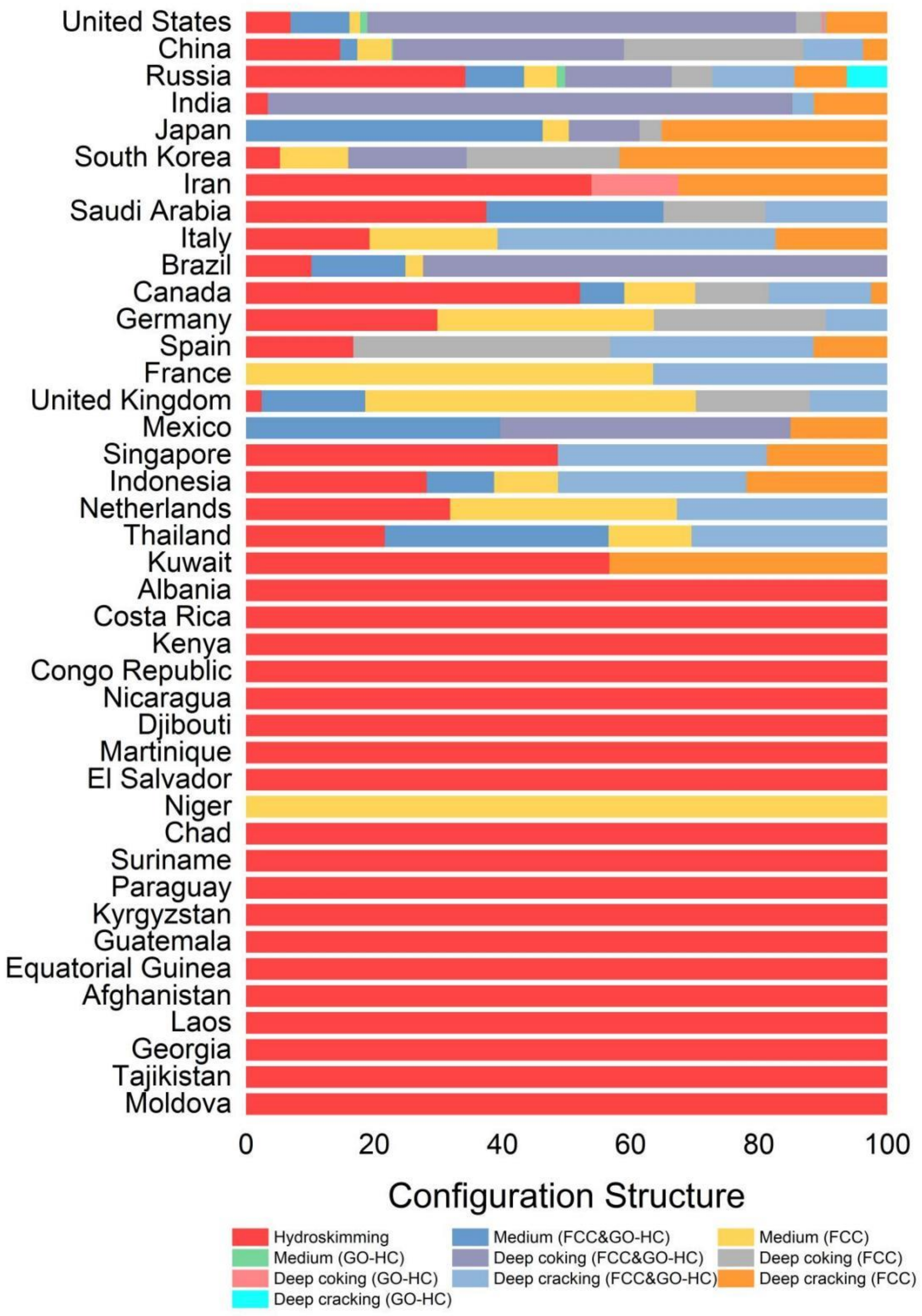
United States
China
Russia
India
Japan
South Korea
Iran
Saudi Arabia
Italy
Brazil
Canada
Germany
Spain
France
United Kingdom
Mexico
Singapore
Indonesia
Netherlands
Thailand
Kuwait
Albania
Costa Rica
Kenya
Congo Republic
Nicaragua
Djibouti
Martinique
El Salvador
Niger
Chad
Suriname
Paraguay
Kyrgyzstan
Guatemala
Equatorial Guinea
Afghanistan
Laos
Georgia
Tajikistan
Moldova
0
20
40
60
80
100
Configuration Structure
Hydroskimming
Medium (FCC&GO-HC)
Medium (FCC)
Medium (GO-HC)
Deep coking (FCC&GO-HC)
Deep coking (FCC)
Deep coking (GO-HC)
Deep cracking (FCC&GO-HC)
Deep cracking (FCC)
Deep cracking (GO-HC)

Figure S6 GHG emissions of important refineries by refining process in other top 20 enterprises

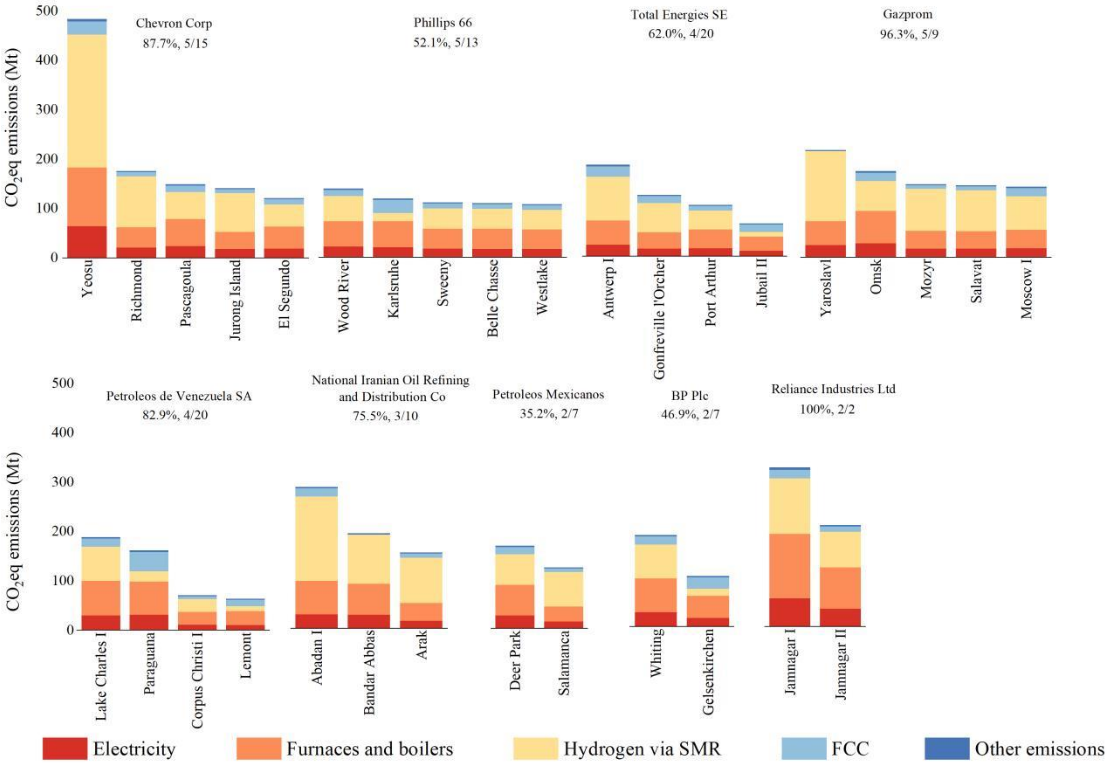

Figure S7 Uncertainty analysis of GHG emissions from global refineries from 2000 to 2021

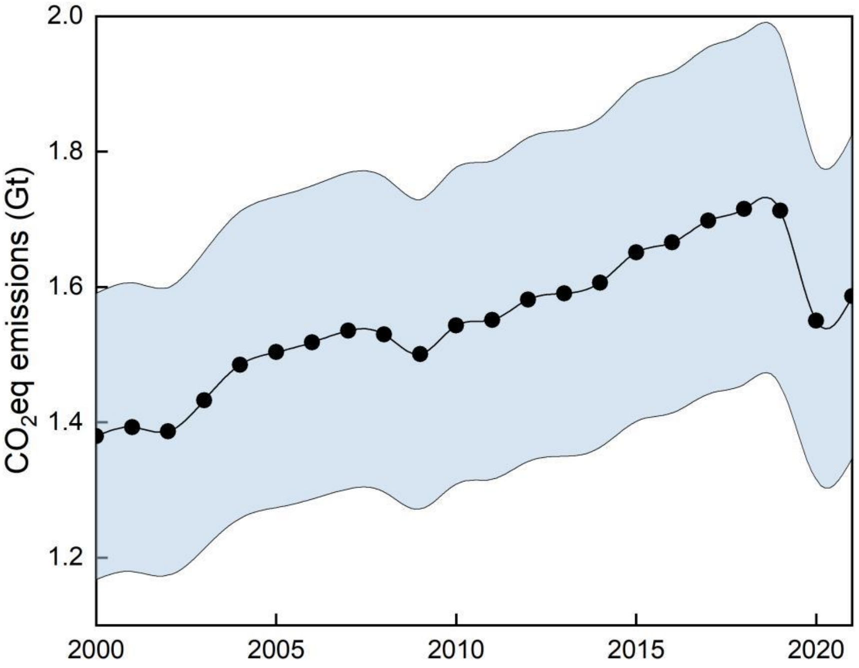

Supplemental Tables

Table S1 Data sources and details of CEADs-GREIv2.0

Data Source	Data Field	Our database information
GlobalData: https://oilgascn.globaldata.com/HomePage	Zone/Country/City<br>Operator<br>Refinery name<br>Refinery status<br>Date of Commissioning<br>Date of Decommissioning<br>Refinery configuration<br>Refining capacity (bbl/d)<br>Main seven shareholder	Zone/Country/City<br>Operator<br>Refinery name<br>Refinery status<br>Date of Commissioning<br>Date of Decommissioning<br>Refinery configuration<br>Refining capacity (bbl/d)<br>Main seven shareholder
A barrel Full: http://abarrelfull.wikidot.com/list-of-global-oil-refineries	Refining units<br>Crude oil supply<br>Refined Products	Crude supply<br>Refined products
Regional, national oil refining industry data: BP: https://www.bp.com/en/global/corporate/energyeconomics/statistical-reviewof-worldenergy/downloads.html	Annual oil refining products production by country	Annual refinery throughput (bbl/d)
Petroleum and other liquids: https://www.eia.gov/international/data/world/petroleum-and-other-liquids	Monthly petroleum and other liquids production (Mb/d)<br>Monthly crude oil including lease condensate production (Mb/d)<br>Monthly NGPL (Mb/d)<br>Monthly other liquids (Mb/d)<br>Monthly refinery processing gain (Mb/d)	Monthly crude oil including lease condensate production (Mb/d)
PRELIM	Crude oil samples<br>Carbon density of different refinery configurations	Crude oil samples<br>Carbon density of different refinery configurations

Table S2 Details of the possible existing process units in each configuration.

Configuration	Process unit
Hydroskimming	Desalter, Atmosphere Tower Furnace, Atmosphere Tower, Naphtha Hydrotreater, Kerosene Hydrotreater, Kerosene Merox Unit, Diesel Hydrotreater and Blending, Reformer, Isomerisation Unit, Gasoline Blending
Medium Conversion (FCC)	The Process Unit of Hydroskimming Refineries, FCC Feed Hydrotreater, FCC and Main Fractionator, Alkylation unit
Medium Conversion (GO-HC)	The Process Unit of Hydroskimming Refineries, Gas Oil Hydrocracker and Fractionator, Alkylation unit
Medium Conversion (FCC&GO-HC)	The Process Unit of Hydroskimming Refineries, FCC Feed Hydrotreater, FCC and Main Fractionator, Gas Oil Hydrocracker and Fractionator, Alkylation unit
Deep Coking (FCC)	The Process Unit of Hydroskimming Refineries, FCC Feed Hydrotreater, FCC and Main Fractionator, Gas Oil Hydrocracker and Fractionator, Alkylation unit, Coker Furnace, Coker, Coker Fractionator, Coker Naphtha Hydrotreater
Deep Coking (GO-HC)	The Process Unit of Hydroskimming Refineries, FCC Feed Hydrotreater, FCC and Main Fractionator, Gas Oil Hydrocracker and Fractionator, Alkylation unit, Coker Furnace, Coker, Coker Fractionator, Coker Naphtha Hydrotreater
Deep Coking (FCC&GO-HC)	The Process Unit of Hydroskimming Refineries, FCC Feed Hydrotreater, FCC and Main Fractionator, Gas Oil Hydrocracker and Fractionator, Alkylation unit, Coker Furnace, Coker, Coker Fractionator, Coker Naphtha Hydrotreater
Deep Hydrocracking (FCC)	The Process Unit of Hydroskimming Refineries, FCC Feed Hydrotreater, FCC and Main

	Fractionator, Gas Oil Hydrocracker and Fractionator, Alkylation unit, Residue Hydrocracker Furnace, Residue Hydrocracker, Residue Hydrocracker Fractionator, Hydrocracker Naphtha Hydrotreater
Deep Hydrocracking (GO-HC)	The Process Unit of Hydroskimming Refineries, FCC Feed Hydrotreater, FCC and Main Fractionator, Gas Oil Hydrocracker and Fractionator, Alkylation unit, Residue Hydrocracker Furnace, Residue Hydrocracker, Residue Hydrocracker Fractionator, Hydrocracker Naphtha Hydrotreater
Deep Hydrocracking (FCC&GO-HC)	The Process Unit of Hydroskimming Refineries, FCC Feed Hydrotreater, FCC and Main Fractionator, Gas Oil Hydrocracker and Fractionator, Alkylation unit, Residue Hydrocracker Furnace, Residue Hydrocracker, Residue Hydrocracker Fractionator, Hydrocracker Naphtha Hydrotreater

Table S3 Cumulative GHG emissions of oil refining enterprises from 2000 to 2021

Enterprise	Country	Rank	GHG Emissions (Mt)
China Petrochemical Corp	China	1	1553.528
China National Petroleum Corp	China	2	1445.516
Exxon Mobil Corp	United States	3	1428.638
Valero Energy Corp	United States	4	1418.899
Saudi Arabian Oil Co	Saudi Arabia	5	1270.252
Royal Dutch Shell Plc	Netherlands	6	1164.017
Marathon Petroleum Corp	United States	7	1120.754
Rosneft Oil Co	Russia	8	1051.236
ENEOS Corp	Japan	9	924.141
Chevron Corp	United States	10	869.692
National Iranian Oil Refining and Distribution Co	Iran	11	842.881
Phillips 66	United States	12	813.608
Petroleo Brasileiro SA	Brazil	13	727.077
TotalEnergies SE	France	14	705.928
Indian Oil Corp Ltd	India	15	643.424
Gazprom	Russia	16	625.828
BP Plc	United Kingdom	17	618.312
Petroleos de Venezuela SA	Venezuela	18	590.494
Petroleos Mexicanos	Mexico	19	533.672
Reliance Industries Ltd	India	20	504.719
PBF Energy Inc	United States	21	504.258
Kuwait Petroleum Corp	Kuwait	22	451.331
Lukoil Oil Co	Russia	23	445.102
PT Pertamina	Indonesia	24	425.521

Idemitsu Kosan Co Ltd	Japan	25	392.576
SK Innovation Co Ltd	South Korea	26	380.357
Oil and Natural Gas Corp Ltd	India	27	338.486
Repsol SA	Spain	28	329.840
Eni SpA	Italy	29	327.798
Koch Industries Inc	United States	30	307.734
Bharat Petroleum Corp Ltd	India	31	280.107
HollyFrontier Corp	United States	32	268.719
Suncor Energy Inc	Canada	33	253.255
Compania Espanola de Petroleos SAU	Spain	34	248.398
Surgutneftegas	Russia	35	248.304
Polski Koncern Naftowy Orlen SA	Poland	36	246.002
GS Energy Corp	Canada	37	243.265
Cenovus Energy Inc	Canada	38	230.915
Cosmo Oil Co Ltd	Japan	39	203.633
Formosa Plastics Group	China	40	189.502
Ecopetrol SA	Colombia	41	184.199
MOL Hungarian Oil and Gas Plc	Hungary	42	178.067
The Bahrain Petroleum Company BSC	Bahrain	43	176.600
CPC Corp	China	44	171.207
Corral Petroleum Holdings AB	Cyprus	45	165.047
China National Offshore Oil Corp	China	46	163.290
Irving Oil Ltd	Canada	47	146.322
LyondellBasell Industries NV	United States	48	144.408
Abu Dhabi National Oil Co	United Arab Emirates	49	140.180
Korea Shipbuilding & Offshore Engineering Co Ltd	South Korea	50	139.572
PTT Public Co Ltd	Thailand	51	139.004
Delta Air Lines Inc	United States	52	133.699

Neste Corp	Finland	53	132.377
OMV AG	Austria	54	131.642
Sonatrach SpA	Algeria	55	130.090
Others Ltd	Turkey	56	119.298
Petroliam Nasional Bhd	Malaysia	57	118.803
National Company KazMunayGas	Kazakhstan	58	114.658
Ineos Ltd	Switzerland	59	113.472
Grupa Lotos SA	Poland	60	111.455
Hellenic Petroleum SA	Greece	61	109.961
YPF SA	Argentina	62	107.678
Others Ltd	Thailand	63	107.375
Oil Refineries Ltd	Israel	64	106.915
Shaanxi Yanchang Petroleum Co Ltd	China	65	105.193
China National Chemical Corp	China	66	100.961
Delek US Holdings Inc	United States	67	99.474
Egyptian General Petroleum Corp	Egypt	68	99.391
Koc Holding AS	Turkey	69	98.712
Motor Oil Corinth Refineries SA	Greece	70	97.722
Equinor ASA	Norway	71	94.099
Others Ltd	Italy	72	93.863
SAFMAR Group	Russia	73	92.650
Refineria di Korsou NV	Cura?ao	74	91.886
ArcLight Capital Partners LLC	United States	75	88.396
Taiyo Oil Co Ltd	Japan	76	85.859
Others Ltd	Belarus	77	85.634
Others Ltd	India	78	85.490
Hilco Redevelopment Partners LLC	United States	79	83.042
Galp Energia SGPS SA	Portugal	80	82.924
Vitol Holding II SA	Luxembourg	81	82.214

Others Ltd	Japan	82	78.430
Empresa Nacional del Petroleo	Chile	83	77.169
Icahn Enterprises LP	United States	84	73.009
Shandong Dongming Petrochemical Group Co Ltd	China	85	72.937
Sinochem Holdings Corp Ltd	China	86	71.925
Gunvor Group Ltd	Cyprus	87	64.312
Energy Transfer LP	United States	88	62.533
Bangchak Corporation PCL	Thailand	89	61.963
Saras SpA	Italy	90	61.663
Essar Energy Ltd	United Kingdom	91	59.968
CHS Inc	United States	92	58.763
National Oil Corporation of Libya	Libya	93	58.189
Trinidad Petroleum Holdings Ltd	Republic Of Trinidad And Tobago	94	57.983
State Oil Company of the Azerbaijan Republic	Azerbaijan	95	57.665
Trafigura Group Pte Ltd	Singapore	96	52.447
Banias Refinery Co	Syria	97	50.679
San Miguel Corp	Philippines	98	48.279
Qatar Petroleum	Qatar	99	47.193
Hunt Consolidated Inc	United States	100	47.132
Others Ltd	Russia	101	46.682
Shandong Hengyuan Petrochemical Co Ltd	China	102	46.588
Tatneft	Russia	103	46.469
Sinclair Oil Corp	United States	104	46.223
Ampol Ltd	Australia	105	45.804
Turkmenbashi Oil Processing Complex	Turkmenistan	106	43.477
Sumitomo Chemical Co Ltd	Japan	107	41.957
Jordan Petroleum Refinery Co Ltd	Jordan	108	41.636
State Oil Ltd	United Kingdom	109	40.735

North Refineries Co	Iraq	110	40.274
Taif	Russia	111	38.238
Baota Petrochemical Group	China	112	37.775
Others Ltd	Saudi Arabia	113	36.755
Lihuayi Group Co Ltd	China	114	34.818
EP Petroecuador	Ecuador	115	34.025
Calumet Specialty Products Partners LP	United States	116	33.493
Naftan	Belarus	117	33.222
Petroplus Holdings AG	Switzerland	118	33.019
Others Ltd	South Africa	119	32.838
ERG SpA	Italy	120	32.561
Others Ltd	Oman	121	30.630
South Refineries Co	Iraq	122	30.195
CVR Energy Inc	United States	123	29.821
Federated Co-operatives Ltd	United States	124	29.479
Cuba Petroleo SA	Cuba	125	29.060
Fujian Petrochemical Industrial Group Company Ltd	China	126	28.346
Par Pacific Holdings Inc	United States	127	28.301
Sasol Ltd	South Africa	128	28.131
Others Ltd	Ukraine	129	28.121
Global Clean Energy Holdings Inc	United States	130	27.608
Canadian Natural Resources Ltd	Canada	131	27.577
Others Ltd	Philippines	132	27.296
Sudanese Petroleum Corp	Sudan	133	27.237
Reggeborgh Groep BV	netherlands	134	26.486
The Carlyle Group Inc.	United States	135	26.486
Api-Anonima Petroli Italiana SpA	Italy	136	26.296
Others Ltd	China	137	25.967

Others Ltd	United States	138	24.233
Nigerian National Petroleum Corp	Nigeria	139	24.171
Glencore Plc	Switzerland	140	23.780
Mittal Investments Sarl	Luxembourg	141	22.692
Paz Oil Co Ltd	Israel	142	22.563
ShanDong Kenli Petrochemical Co Ltd	China	143	22.462
Shandong Hi-Tech Chemical Group Co Ltd	China	144	22.151
Freepoint Commodities LLC	United States	145	22.099
Oil India Ltd	India	146	21.622
Vietnam National Oil and Gas Group	Vietnam	147	21.290
Red Apple Group Inc	United States	148	19.324
Cosan Distribuidora de Combustiveis Ltda	Brazil	149	19.071
The New Zealand Refining Co Ltd	New Zealand	150	18.970
Midland Refineries Co	United States	151	18.810
Placid Refining Co LLC	United States	152	18.756
Greenergy International Ltd	United Kingdom	153	18.527
Shandong Haiyou Petrochemical Group Co Ltd	China	154	17.832
Aden Refinery Co	United Kingdom	155	17.320
Grampet SA	Romania	156	17.232
Others Ltd	South Korea	157	16.975
Shandong Shenchi Chemical Co Ltd	China	158	16.717
Emirates National Oil Co Ltd	United Arab Emirates	159	16.066
Calcasieu Refining Co	United States	160	15.966
Others Ltd	Germany	161	15.880
SK Inc	South Korea	162	15.635
Bitumina Industries Ltd	United Kingdom	163	15.533
Canadian Oil Sands Ltd	Canada	164	15.424
Naftogaz of Ukraine	Ukraine	165	15.421

National Iranian Oil Co	Iran	166	15.215
Plains All American Pipeline LP	United States	167	15.128
Klesch & Co Ltd	United Kingdom	168	15.127
Zhejiang Rongsheng Holding Group Co Ltd	China	169	15.103
Shandong Chenxi Petrochemical Co Ltd	China	170	15.081
OQ SAOC	Oman	171	14.910
Dongying Petroleum Chemical Co Ltd Hualian	China	172	14.495
Sharjah Oil Refining Co Fzc	United Arab Emirates	173	14.349
Silverpeak Strategic Partners LP	United States	174	14.137
Others Ltd	Pakistan	175	14.134
Arctic Slope Regional Corp	United States	176	13.892
Others Ltd	Romania	177	13.774
Shandong Binhua Group Co Ltd	China	178	13.309
Neftepererabotka	Russia	179	13.132
Penglai Ampang Petrochemical Co Ltd	China	180	13.110
Shandong Dongfang Hualong Industry and Trading Co Ltd	China	181	12.888
Persian Gulf Star Oil Co	Iran	182	12.743
Sonangol EP	Angola	183	12.680
St1 Oy	Finland	184	12.481
Homs Refinery Co	Syria	185	12.320
World Energy LLC	United States	186	12.113
TRASTA Energy Ltd	Libya	187	11.829
Qianhai Petroleum & Chemical Group Co Ltd	China	188	11.799
Guangrao Kelida Petrochemical	China	189	11.643
China North Industries Corp	China	190	11.590
Uzbekneftegaz National Holding Co	Uzbekistan	191	11.251
Ergon Inc	United States	192	11.213
Buckeye Partners LP	United States	193	11.067

Mitsubishi Corp	Japan	194	11.062
Shandong Jingbo Petrochemical Co Ltd	China	195	10.894
Others Ltd	Spain	196	10.705
PL ESG Denmark Co ApS	Denmark	197	10.553
Shandong Qingyuan Petrochemical Co Ltd	China	198	10.509
Others Ltd	Morocco	199	10.142
Parkland Corp	Canada	200	9.527
NK RussNeft	Russia	201	9.426
Hudson Oil Corporation Ltd	Canada	202	9.163
Slavyansk ECO	Russia	203	8.833
Novatek	Russia	204	8.595
Hengli Petrochemical Co Ltd	China	205	8.440
Shandong Shangneng Group	China	206	8.431
Petroperu SA	Peru	207	8.230
Shandong Shengkai Petrochemical Co Ltd	China	208	8.001
CountryMark Cooperative Holding Corp	United States	209	7.908
Sichuan Shengma Chemical Industry Stock Co Ltd	China	210	7.891
San Joaquin Refining Co Inc	United States	211	7.868
Administracion Nacional de Combustibles, Alcoholes y Portland	Uruguay	212	7.746
Gaoqing Hongyuan Petrochemical Co Ltd	China	213	7.709
FJ Management Inc	United States	214	7.670
The Attock Oil Co Ltd	Pakistan	215	7.589
Koninklijke Vopak NV	Netherlands	216	7.571
Shandong Jincheng Petrochemical Group Co Ltd	China	217	7.546
Tema Oil Refinery Ltd	Ghana	218	7.498
Oil Combustibles SA	Argentina	219	7.395
Bridas Energy Holdings Ltd	Argentina	220	7.367
Others Ltd	North Korea	221	7.317

Shouguang Lianmeng Petrochemical Co Ltd	China	222	7.134
Yug Energo	Russia	223	7.087
Shandong Huifeng Petroleum & Chemical Group Co Ltd	China	224	7.056
China Offshore Oil & Gas Development & Utilization Co	China	225	6.918
Gulf Atlantic Operations LLC	United States	226	6.831
Blue Star Daqing Petroleum Co Ltd	China	227	6.676
Liquid Petroleum Development Co	South Africa	228	6.581
Petroleum Corporation of Jamaica	Jamaica	229	6.193
Jihua Group Corporation Ltd	China	230	5.923
Tongkun Group Co Ltd	China	231	5.923
Yacimientos Petroliferos Fiscales Bolivianos	Bolivia	232	5.847
Monument Chemical LLC	United States	233	5.826
Pakistan State Oil Co Ltd	Pakistan	234	5.694
Gaetano LLC	United States	235	5.626
KNGK Group	Russia	236	5.596
Kinder Morgan Inc	United States	237	5.575
Belvor Holdings Ltd	Cyprus	238	5.560
Shandong Befar Group Binyang Fuel Chemical Co Ltd	China	239	5.533
Shandong Yuhuang Chemical Co Ltd	China	240	5.522
Shanghai Chemical Industry Group Co Ltd	China	241	5.515
Lanaz Co	Iraq	242	5.447
Shandong Shida Technology Group Co Ltd	China	243	5.390
IPLOM SpA	Italy	244	5.376
HaiKe Chemical Group Ltd	China	245	5.307
Mubadala Investment Co	United Arab Emirates	246	5.297
Zhuhai Port Co Ltd	China	247	5.294

Hanwha Corp	South Korea	248	5.096
Others Ltd	Cameroon	249	5.070
Dididom Petroleum Holding	Bulgaria	250	5.069
Others Ltd	United Kingdom	251	5.065
Byco Busient Incorporated	United Kingdom	252	4.915
Shandong Wantong Petrochemical Group Co Ltd	China	253	4.896
Young Refining Corp	United States	254	4.836
Silver Eagle Refining Inc	United States	255	4.792
Others Ltd	Malaysia	256	4.716
Myanma Petrochemical Enterprise	Myanmar	257	4.703
Blue-star Petrochemical Co Ltd	China	258	4.672
Kern Oil & Refining Co	United States	259	4.574
KAR Oil Refining Ltd	Iraq	260	4.477
NefteChemService	Russia	261	4.418
Changchun Xinda Petroleum Group Co Ltd	China	262	4.416
Mari Oil Refinery	Republic of Belarus	263	4.394
SHENXIAN Huaxiang Petrochemical Co Ltd	China	264	4.331
Dongying Qirun Chemical Co Ltd	China	265	4.299
H&R GmbH & Co KGaA	Germany	266	4.185
Societe Nationale d'Operations Petrolieres de la Cote d'Ivoire	Cote d'Ivoire	267	4.084
Bangladesh Petroleum Corp	Bangladesh	268	4.036
IFM Investors Pty Ltd	Australia	269	3.891
Yingkou Jiafu Petrochemical Co Ltd	China	270	3.708
Foreland Refining Corp	United States	271	3.694
Societe Tunisienne des Industries de Raffinage	Tunisia	272	3.656
Guangdong Tianyi Group Co Ltd	China	273	3.610
Others Ltd	Venezuela	274	3.537
Sudapet Co Ltd	Sudan	275	3.530

Ceylon Petroleum Corp	Sri Lanka	276	3.505
Ube Industries Ltd	Japan	277	3.311
Abraaj Capital Ltd	United Arab Emirates	278	3.277
Boxing County, Shandong Province Yongxin Chemical Co Ltd	China	279	3.160
Others Ltd	Egypt	280	3.116
Panlong Petrochemical Co Ltd	China	281	2.964
Jinhai Hongye Petrochemical Co Ltd	China	282	2.953
Others Ltd	Indonesia	283	2.924
Starlight Relativity Acquisition Co LLC	United States	284	2.867
MFC Capital	United States	285	2.816
Jindayuan Real Estate Co Ltd	China	286	2.816
Xindu Group Co Ltd	China	287	2.816
Hebei Xinquan Petroleum & Chemical Co Ltd	China	288	2.736
SC Condensate	Kazakhstan	289	2.716
ConocoPhillips	United States	290	2.711
Ruifeng Petroleum Chemical Holdings Ltd	China	291	2.666
Tipco Asphalt Public Company Limited	Thailand	292	2.647
Omnimpex Chemicals SA	Romania	293	2.576
Industrial Development Corporation Ltd	South Africa	294	2.556
Li & Fung Ltd	China	295	2.546
Shandong Tianhong Energy Chemical Co Ltd	China	296	2.463
Refinadora Costarricense de Petroleo	Costa Rica	297	2.411
Guangzhou HuaHong Oil Co Ltd	China	298	2.409
Qihua Group Co Ltd	China	299	2.409
Zarubezhneft	Russia	300	2.407
Government of Kenya	Kenya	301	2.390
Others Ltd	Switzerland	302	2.381
Others Ltd	Poland	303	2.363

PetroChina Jiangsu Xingneng Asphalt Co Ltd	China	304	2.308
JX Nippon Mining & Metals Corp	United States	305	2.249
State-owned Assets Supervision and Administration Commission of the State Council	China	306	2.236
Refinaria de Petroleos de Manguinhos SA	Mexico	307	2.230
Others Ltd	Sweden	308	2.178
Hebei Dagang PetroChemical Co Ltd	China	309	2.165
Yenisey	Russia	310	2.152
Societe Nationale des Petroles du Congo	Congo Republic	311	2.151
Others Ltd	Sudan	312	2.132
Tidewater Midstream and Infrastructure Ltd	United States	313	2.079
Lazarus Energy Holdings LLC	United States	314	2.058
Uniper SE	Germany	315	2.048
Gibson Energy Inc	United States	316	2.031
Wudi Xin Yue Chemical Co Ltd	China	317	2.029
Hilcorp Energy Co	United States	318	2.024
Destileria Argentina De Petroleo SA	Argentina	319	2.015
Magellan Midstream Partners LP	United States	320	1.974
WSP Krutogorsky Refinery	Russia	321	1.818
Hebei Refinery Huayu Bitumen Products Co Ltd	China	322	1.808
Heilongjiang Haiguolong Oil and Petrochemical Co Ltd	China	323	1.797
Shaanxi Shuangyi Petrochemical Co Ltd	China	324	1.797
Unity strength Co Ltd	China	325	1.797
PetroNeft Resources Plc	United States	326	1.769
American Refining Group Inc	United States	327	1.766
Jiyang Guangyuanfa Asphalt Co Ltd	China	328	1.750
HCS Group GmbH	Germany	329	1.749
Rafinaria Darmanesti SA	Romania	330	1.737
Gabonese Republic	Gabon	331	1.723

Others Ltd	Slovakia	332	1.719
Greka Oil and Gas Inc	United States	333	1.694
World Oil Corp	United States	334	1.694
Foshan Sanshui Futeng Asphalt Co Ltd	China	335	1.693
Al Brooge Securities Co	United Arab Emirates	336	1.677
Government of the Commonwealth of Dominica	Dominica	337	1.672
Qaiwan Group	Iraq	338	1.670
Tomsk Refining AB	Russia	339	1.645
Tosk Energji	Albania	340	1.639
Qinhuangdao PetroChina Petrochemical Co Ltd	China	341	1.637
Qinhuangdao Yuandong Petroleum Refinery Co Ltd	China	342	1.565
Liaoning Huayou Petrochemical Co Ltd	China	343	1.563
Falconbridge Dominicana SA	Dominica	344	1.543
Zhejiang Hengyi Group Co Ltd	China	345	1.533
Petromax Refining Co LLC	United States	346	1.533
Ventura Refining & Transmission LLC	United States	347	1.476
Government of Ukraine	Ukraine	348	1.474
Parkland Industries Limited	Niger	349	1.446
Dongguan Yelian Petrochemical Co Ltd	China	350	1.445
Hangzhou Xinya Petrochemical Co Ltd	China	351	1.445
Hebei Xinhai Chemical Group Co Ltd	China	352	1.439
Jiangsu Lingguang Co Ltd	China	353	1.368
Pelican Refining Co LLC	United States	354	1.364
Guangzhou Jiasheng Asphalt Co Ltd	China	355	1.361
Pluspetrol Resources Corp NV	Argentina	356	1.351
Foshan Sanshui Haishengda Road Material Co Ltd	China	357	1.318
Ecodiesel SRL	Romania	358	1.286
Martin Midstream Partners LP	Netherlands	359	1.283

Yingchang Heavy Road Asphalt Co Ltd	China	360	1.266
New Bright International Development Ltd	China	361	1.243
Others Ltd	Cote d'Ivoire	362	1.243
Petrosen	United Kingdom	363	1.226
Ultra Group Of Companies, Inc.	United States	364	1.224
RPCG Public Co Ltd	Thailand	365	1.213
Panjin Xingda Group Co Ltd	China	366	1.199
HBOil	Mongolia	367	1.161
Rubis SCA	France	368	1.153
VPK-Oil	Russia	369	1.143
Liaoning Deying PetroChemical Group Co Ltd	China	370	1.141
Engineers India Ltd	India	371	1.123
Pampa Energia SA	Argentina	372	1.105
Taizhou Dongtai PetroChemical Co Ltd	China	373	1.087
Henan Beili Petrochemical Holding Co Ltd	China	374	1.083
Jinao Science & Technology Chemical Industry Co Ltd	China	375	1.083
Yemen Oil Refinery Co	Yemen	376	1.082
Kreyton Ltd	Romania	377	1.070
Lingang Huarun Asphalt Co Ltd	China	378	1.059
Others Ltd	Senegal	379	1.039
Somerset Oil Inc	United States	380	1.021
The Siam Cement Public Co Ltd	Thailand	381	0.975
Others Ltd	Canada	382	0.963
Others Ltd	United Arab Emirates	383	0.949
Linn Energy Inc	United States	384	0.936
Sunshine Asphalt Chemical Co Ltd	China	385	0.911
Yancheng Lianfu PetroChemical Co Ltd	China	386	0.902
Goodway Refining LLC	United States	387	0.847

Hong Kong Bora Holdings Co Ltd	China	388	0.843
Shengyang Jingfa Bitumen Co Ltd	China	389	0.843
Volkhov-Eco	Brazil	390	0.824
Panjin Angyou Asphalt Co Ltd	China	391	0.820
AL Global Oil JSC	Russia	392	0.820
Staatsolie Maatschappij Suriname NV	Suriname	393	0.805
Others Ltd	Bosnia and Herzegovina	394	0.802
Hartree Partners LP	United States	395	0.797
ENAR Petrotech Services Pvt Ltd	Pakistan	396	0.781
Urals Energy PCL	Cyprus	397	0.776
Marubeni Corp	Japan	398	0.776
Mitsui & Co Ltd	Japan	399	0.776
Noble Technologies Ltd	Russia	400	0.774
Others Ltd	Gabon	401	0.774
Maple Energy Plc	Peru	402	0.752
Petroleos Paraguayos SA	Paraguay	403	0.723
Hainan CNOOC Gas Co Ltd	China	404	0.723
Dalian Haichang Group Co Ltd	China	405	0.719
Liaoning Panjin Petrochemical Co Ltd	China	406	0.719
Damai Holdings Ltd	Singapore	407	0.657
Panjin Liaotong Chemical Co Ltd	China	408	0.656
PT Tri Wahana Universal	Indonesia	409	0.639
Xianglu Petrochemicals Co Ltd	China	410	0.588
Others Ltd	Papua New Guinea	411	0.577
Government of Brunei Darussalam	Brunei	412	0.553
Niger Government	Niger	413	0.505
Changle Huarong Industry and Trade Co Ltd	China	414	0.493
Ocyan	France	415	0.482

Perenco Holdings	United Kingdom	416	0.482
Liaozhong Refinery Co Ltd	China	417	0.481
Hebei Refinery Huayou Paraffin Co Ltd	China	418	0.480
The Sol Group	United Kingdom	419	0.471
Saigon Petro Co Ltd	Vietnam	420	0.455
Kat Group	China	421	0.452
Others Ltd	Singapore	422	0.446
Vernal Oil Kazakhstan	Kazakhstan	423	0.445
Others Ltd	Nicaragua	424	0.444
Uralneft Management Co	Russia	425	0.438
Chernigov Refinery	Russia	426	0.426
Iraq National Oil Co	Iraq	427	0.424
Peyman Ramshir Polymer Co	Iran	428	0.421
Piroozi Oil and Gas Refinery Co	Iran	429	0.408
Mitsui Chemicals Inc	Japan	430	0.406
Shandong Donghao Petrochemical Co Ltd	China	431	0.404
Inpex Corp	Japan	432	0.389
Others Ltd	El Salvador	433	0.366
Panjin Dongfang Asphalt Coking Co Ltd	China	434	0.360
Panjin Taipinghe Asphalt Co Ltd	China	435	0.360
GP Global	United Arab Emirates	436	0.347
Nam Viet Refinery JSC	Vietnam	437	0.347
Societe des Hydrocarbures du Tchad	Chad	438	0.335
Others Ltd	Brazil	439	0.334
Kyrgyzneftegaz	Kyrgyzstan	440	0.328
California Resources Corp	United States	441	0.327
Others Ltd	Equatorial Guinea	442	0.325
Super Refinery Pvt Ltd	Bangladesh	443	0.322

Viaro Energy Ltd	United Kingdom	444	0.313
Bangladesh Oil, Gas and Mineral Corp	Bangladesh	445	0.311
DNO ASA	Norway	446	0.301
Refisur SA	Mexico	447	0.289
Oro Negro Refineria SA	bolivia	448	0.272
Slate Refining LLC	United States	449	0.270
Roham Sperlus Co	Iran	450	0.269
Aulac Corp	Vietnam	451	0.243
Hebei Jinrui Petroleum Chemical Co Ltd	China	452	0.240
Jieyang Kangda Chemical Industry Co Ltd	China	453	0.232
Turcas Petrol AS	Turkey	454	0.226
Sahara Energy Ltd	United States	455	0.213
Panjin Dongwang Asphalt Co Ltd	China	456	0.211
Kondinsky Refinery	Russia	457	0.210
IOR Group Ltd	United Kingdom	458	0.201
Ecomar Energy Solutions FZC	United Arab Emirates	459	0.201
Mingyuan Chemical Co Ltd	China	460	0.193
Reficruz Srl	bolivia	461	0.193
Qalaa Holdings SAE	Egypt	462	0.192
Oil & Gas Development Co Ltd	Pakistan	463	0.183
Jund China Petroleum Co	China	464	0.183
Dong Phuong Petroleum JSC	Vietnam	465	0.176
Shanghai Tonva PectroChemical Co Ltd	China	466	0.175
Suzhou Ruixin Highway Material Co Ltd	China	467	0.175
Others Ltd	Laos	468	0.171
Kam International Oil	Afghanistan	469	0.139
Others Ltd	Kyrgyzstan	470	0.119
Petromax Refinery Ltd	United States	471	0.115

Ghazanfar Group	United Arab Emirates	472	0.114
Others Ltd	Turkmenistan	473	0.114
Platon Gas Oil Ghana Ltd	Ghana	474	0.107
Azimuth	Luxembourg	475	0.097
Others Ltd	Argentina	476	0.090
Sichuan Road & Bridge Co Ltd	China	477	0.088
Xinjiang International Industry Co Ltd	China	478	0.083
ZD Oil Co Ltd	Georgia	479	0.083
Centrex Energy & Gas AG	Austria	480	0.082
RTS Oil	United States	481	0.066
Chengdu Road & Bridge Engineering Co., Ltd.	China	482	0.063
Aqua Refinery Ltd	Bangladesh	483	0.060
Behin Aras Distillation Co	Iran	484	0.057
Ansar Oil Refinery	United States	485	0.045
Lao State Fuel Co	Laos	486	0.043
CVO Petrochemical Refinery Ltd	Bangladesh	487	0.040
Dome Energy AB	United States	488	0.034
Khasan and Co	Russia	489	0.029
BITEX Refinery	Albania	490	0.028
Others Ltd	Tajikistan	491	0.025
Pampetrol SAPEM	Argentina	492	0.023
Sakhalin Oil Co	Russia	493	0.019
Azpetrol Ltd	Azerbaijan	494	0.014
Naftrason	Tajikistan	495	0.013
Others Ltd	Nigeria	496	0.012
Mehron Oil	Tajikistan	497	0.006
Petrolin Group	United Kingdom	498	0.001

Table S4 Detailed information of top 20 countries

Enterprises	Country	National oil company
Petroleo Brasileiro SA	Brazil	Yes
China National Petroleum Corp	China	Yes
China Petrochemical Corp	China	Yes
TotalEnergies SE	France	No
Indian Oil Corp Ltd	India	Yes
Reliance Industries Ltd	India	No
National Iranian Oil Refining and Distribution Co	Iran	Yes
ENEOS Corp	Japan	No
Petroleos Mexicanos	Mexico	Yes
Royal Dutch Shell Plc	Netherlands	No
Gazprom	Russia	No
Rosneft Oil Co	Russia	No
Saudi Arabian Oil Co	Saudi Arabia	Yes
BP Plc	United Kingdom	No
Chevron Corp	United States	No
Exxon Mobil Corp	United States	No
Marathon Petroleum Corp	United States	No
Phillips 66	United States	No
Valero Energy Corp	United States	No
Petroleos de Venezuela SA	Venezuela	Yes

Table S5 Cumulative GHG emissions of top 20 countries

Countries	Region	Hydroskimming	Medium conversion	Deep coking	Deep hydrocracking	Cumulative GHG emissions (Mt)	Share of the regional cumulative GHG emissions (%)	Share of the global cumulative GHG emissions (%)	Carbon intensity (kg/bbl)
Thailand	India and ASEAN	2	3	0	2	383.60	10.84	1.12	49.8
Netherlands	EU 27 & UK	3	2	0	1	429.53	8.47	1.26	46.8
Indonesia	India and ASEAN	7	2	0	2	430.06	12.15	1.26	59.3
Singapore	India and ASEAN	3	0	0	2	448.92	12.68	1.32	57.8
Mexico	Other Americas	0	2	3	1	457.61	17.01	1.34	53.0
United Kingdom	EU 27 & UK	4	6	1	1	458.39	9.04	1.34	42.9
France	EU 27 & UK	0	9	0	3	539.62	10.64	1.58	50.2
Spain	EU 27 & UK	3	0	4	3	593.04	11.69	1.74	62.3
Germany	EU 27 & UK	7	5	2	1	656.21	12.94	1.92	41.1

Canada	Other Americas	12	6	3	6	697.23	25.92	2.04	50.0
Brazil	Other Americas	5	4	7	0	731.35	27.19	2.14	51.5
Italy	EU 27 & UK	8	2	1	6	745.38	14.70	2.18	60.1
Saudi Arabia	Middle East & North Africa	6	2	1	2	783.68	22.99	2.30	46.0
Iran	Middle East & North Africa	13	0	1	3	856.78	25.14	2.51	58.0
South Korea	Asia & Oceania	1	1	2	2	1461.28	42.55	4.28	71.7
Japan	Asia & Oceania	6	13	4	9	1712.21	49.86	5.02	60.1
India	India and ASEAN	4	0	15	4	2004.03	56.63	5.87	67.1
Russia	Other Europe and CIS	44	6	6	7	2206.51	76.82	6.47	55.8
China	China	72	50	65	17	4300.31	100.00	12.60	56.7
United States	United States	53	32	61	19	8222.18	100.00	24.10	66.8

Table S6 Crude oil classification based on API gravity and sulfur content[1]

Type	API gravity (°)	Sulfur content (wt%)	Default refinery configuration
Light crude oil	>32	⩽0.5, Sweet Light	Hydroskimming
	>32	⩾0.5, Sour Light	Medium conversion
Medium crude oil	22-32	⩽0.5, Sweet Medium	Medium conversion
	22-32	⩾0.5, Sour Medium	Medium conversion
Heavy crude oil	⩽22	⩽0.5, Sweet Heavy	Deep conversion
	⩽22	⩾0.5, Sour Heavy	Deep conversion

Table S7 Comparison between the global refining industry GHG emissions estimated by this work and previous studies

	Year	GHG emissions from refineries	Countries	The number of operating refineries	Refined oil production (Mbd)	Refining capacity (Mbd)
Jing et al. (2020)[1]	2015	1.2 Gt	83	478	74.4	-
Lei et al. (2021)[2]	2018	1.3 Gt	-	946	-	98
This Study	2015	1.65 Gt	121	1095	80.1	-
This Study	2018	1.72 Gt	121	1095	-	105

Table S8 Default Settings for the PRELIM model

Items	Default setting
Naphtha catalytic reformer	SR Naphtha
FCC hydrotreater options	Post-hydrotreater
Electricity source	Coal fired
SMR hydrogen purification options	Amine CO2 removal
Allocation method	Mass Basis
Heating value	Lower heating Values (LHV)
Global warming potential	2013 IPCC AR5 (100 years) [default]
Upstream Releases	Include
Off-site Managed Waste Releases	Include
Offgas Product Production	off
Cogeneration Unit	No Cogeneration Unit

Reference

1. Jing L., El-Houjeiri H. M., El-Houjeiri J.-C., et al. (2020). Carbon intensity of global crude oil refining and mitigation potential. Nat. Clim. Change 10, 526-532.
2. Lei T., Guan D., Shan Y., et al. (2021). Adaptive CO2 emissions mitigation strategies of global oil refineries in all age groups. One Earth 4, 1114-1126.
